# Review of Chromatographic Methods Coupled with Modern Detection Techniques Applied in the Therapeutic Drugs Monitoring (TDM)

**DOI:** 10.3390/molecules25174026

**Published:** 2020-09-03

**Authors:** Tomasz Tuzimski, Anna Petruczynik

**Affiliations:** 1 Department of Physical Chemistry, Medical University of Lublin, Chodźki 4a, 20-093 Lublin, Poland; 2 Department of Inorganic Chemistry, Medical University of Lublin, Chodźki 4a, 20-093 Lublin, Poland

**Keywords:** therapeutic drug monitoring (TDM), liquid chromatography, detection techniques, biological samples, sample preparation

## Abstract

Therapeutic drug monitoring (TDM) is a tool used to integrate pharmacokinetic and pharmacodynamics knowledge to optimize and personalize various drug therapies. The optimization of drug dosing may improve treatment outcomes, reduce toxicity, and reduce the risk of developing drug resistance. To adequately implement TDM, accurate and precise analytical procedures are required. In clinical practice, blood is the most commonly used matrix for TDM; however, less invasive samples, such as dried blood spots or non-invasive saliva samples, are increasingly being used. The choice of sample preparation method, type of column packing, mobile phase composition, and detection method is important to ensure accurate drug measurement and to avoid interference from matrix effects and drug metabolites. Most of the reported procedures used liquid chromatography coupled with tandem mass spectrometry (LC-MS/MS) techniques due to its high selectivity and sensitivity. High-performance chromatography with ultraviolet detection (HPLC-UV) methods are also used when a simpler and more cost-effective methodology is desired for clinical monitoring. The application of high-performance chromatography with fluorescence detection (HPLC-FLD) with and without derivatization processes and high-performance chromatography with electrochemical detection (HPLC-ED) techniques for the analysis of various drugs in biological samples for TDM have been described less often. Before chromatographic analysis, samples were pretreated by various procedures—most often by protein precipitation, liquid–liquid extraction, and solid-phase extraction, rarely by microextraction by packed sorbent, dispersive liquid–liquid microextraction. The aim of this article is to review the recent literature (2010–2020) regarding the use of liquid chromatography with various detection techniques for TDM.

## 1. Introduction

The health of the human population is largely determined by the effectiveness of the therapies used as well as the side effects of pharmaceuticals that can threaten the safety of people. Patients often are treated with a fixed drug dose, without considering the possible simultaneous coadministration of other drugs or genetic and environmental factors altering the absorption, distribution, metabolism, and excretion of the drug. Therefore, more accurate detection and monitoring of the drug’s concentrations in the organisms is necessary. 

Therapeutic drug monitoring (TDM) is a multidisciplinary clinical practice used for the optimization and individualization of drug therapy in the general and special populations that has been predominantly used to prevent or minimize adverse events produced by drugs, especially with a narrow therapeutic index [1]. A defined therapeutic target range (therapeutic window) is an essential requirement to optimize pharmacotherapy by TDM. If the drug level stays within this range, a therapeutic response combined with good tolerability can most likely be assumed. If the measured value falls below the therapeutic target range, there is a high probability that the response to treatment does not significantly differ from placebo. TDM represents a strategy to personalize the therapy by tailoring the dose for the patient, which is fundamental with many drugs, especially drugs with a narrow therapeutic window. TDM helps improve treatment efficacy and safety. To effectively perform TDM, a dependency between drug concentration (pharmacokinetics) and pharmacological effect (pharmacodynamics), which can be efficacy and/or toxicity, needs to have been established [2]. The TDM results can be used to assist in the determination of whether the patient has developed viral resistance to the prescribed drugs or whether the patient has simply stopped taking the drugs. TDM is also helpful in determining whether the drug is properly absorbed. The TDM results can be also applied to assist in the determination of whether the patient treated by drugs such as antibiotics, antiviral, or antifungal acquired resistance to the prescribed drugs or whether the patient has stopped taking the drugs. 

The main indications for the application of TDM are as follows: abnormal response to the therapy, unexpected toxicity, suspected chronic abuse, lack of adherence, self-medication, taking other medications, changes in liver or renal function, those with altered metabolism (pregnant women, children, elder, and obese, among others), administration of drugs with a narrow therapeutic range, observation of a poor correlation dosage–clinical response, a well-established relationship between the plasmatic concentration and the clinical effects, observed side effects similar to the symptoms of the disease, the impossibility of monitoring the evolution of the patient by physical examination or common biochemical analysis, possible associated toxicity, and previously noticed inter- and intra-individual variability in the metabolization and clinical effects [3].

TDM is carried out through the quantification of the drug, and often, its main metabolites in physiological fluids (e.g., plasma, serum, and urine) at several times after the drug administration and at several stages of the treatment. For most systemic therapies, drug concentration in blood (or plasma) is recognized as the leading factor associated with pharmacological or toxic effects. Therefore, the majority of assays utilize plasma, rarely whole blood, as the bioanalytical matrix. As intracellular drug concentrations may be more reflective of drug efficacy than that of plasma or whole blood, human peripheral blood mononuclear cells were also sometimes used as a sample matrix for TDM. Dried blood spots, offering the dual benefits of being minimally invasive and requiring minute blood volumes, have also been rarely utilized for drug concentrations determination. Urine, less hair, and various tissues are also rarely employed for TDM.

The implementation of TDM for the clinical management of specific drug therapy requires the availability of suitable bioanalytical methodologies to support the specific drug concentration measurements in the biological samples of interest in order to adjust the patient’s medication regimen and achieve optimal therapeutic outcomes. The following criteria are necessary for drugs to be suitable for TDM: a relationship should exist between the dose and biological samples (most often blood) concentration and between the biological samples concentration and the therapeutic effect of the drug; a narrow therapeutic index; inter-individual pharmacokinetic variability or a poor relationship between the dose and drug response; the pharmacological response should be difficult to assess or distinguish from the adverse effects; and the result of TDM testing must be interpretable and actionable—there should be an effect on clinical outcomes. There are many drugs that meet most of the criteria for TDM but for which measurement of their concentrations is not still commonly applied. Many drugs are bound to serum proteins, but only the free (unbound) drug is responsible for the pharmacological activity of the drug. Most often, the total drug concentration (bound drug and free drug) is measured for the purpose of TDM. However, for certain strongly protein-bound drugs, monitoring the free drug concentration may be necessary for particular patients.

TDM strategies are increasingly reliant on precise and unbiased analytical methods, especially for low sample concentrations. Additionally, automation, high-throughput instruments, robustness, and low costs are prerequisites for selecting a suitable analytical method. TDM has been applied for many years using immunoassay, but it is recognized that immunoassay methods can suffer non-specific interference from related compounds, metabolite interference, or matrix effects. Currently, increasingly reliable, sensitive, and high-quality analytical methods such as liquid chromatography methods coupled with UV, fluorescence detection (FLD), or MS detectors were the main techniques used for TDM. The ability of liquid chromatography to separate individual compounds from other drugs and metabolites present in the biological matrix, combined with selective detection techniques, provides high sensitivity and specificity. The choice of sample preparation method, column technology, internal standard, and detection conditions is important to ensure appropriate drug measurement and avoid interference from matrix effects and drug metabolites [4]. The validation process should include optimization of the analyte, retention of the column, and the demonstration of clean chromatograms with no isobaric interference. After the administration of many drugs, these compounds reach a good concentration in biological fluids, and thus, the sensitivity does not represent an analytical problem. In these situations, a UV detector can be successfully used, as long as the selectivity is properly evaluated by considering possible interference from the coadministrated medications and components of the sample matrix. In recent years, liquid chromatography coupled with mass spectrometry (LC-MS) or tandem mass spectrometry (LC-MS/MS) is increasingly utilized in drug analysis and now is considered to be the gold standard analytical method in TDM. LC-MS or especially LC-MS/MS are increasingly important tools in TDM as they offer increased sensitivity and specificity compared to other methods, and they may be the only viable method for quantifying drugs belonging to various classes. LC-MS significantly reduces the risk of co-eluting substances contributing to the peak area, which is particularly important at lower concentrations of investigated drugs. 

In recent years, many review articles on analytical methods including liquid chromatography for TDM have been published. Most of them discuss methods for a selected, often narrow group of drugs. The application of LC-MS/MS for TDM of various anti-infective drugs was reviewed [5]. The authors focused on the bioanalytical hurdles related to the measurement of anti-infective drugs and on pre- and post-analytical issues. Caro et al. described a review of bioanalytical methods for the therapeutic drug monitoring of β-lactam antibiotics in critically ill patients [1]. Analytical methods useful for the quantitation of statins and their metabolites in biological samples have been summarized and discussed by Patel and Kothari [6]. In 2019, researchers published a review article on the application of LC-MS/MS procedures for TDM of anti-tuberculosis drugs [2]. Zheng and Wang reviewed advances in the application of LC-MS for the determination of antifungal drugs in biological samples [7]. The application of LC-MS/MS for TDM of immunosuppressive drugs was described and discussed in a review article published in 2016 [8]. In the same year, a review article on various analytical methods (immunoassays, LC and MS) for TDM of immunosuppressive drugs was also published [9]. The authors compared analytical methods used for the determination of these drugs in terms of their advantages and disadvantages. Milosheska et al. reviewed the application of dried blood spots for the monitoring and individualization of antiepileptic drug treatment [10]. They discussed advantages, restrictions, and key technical aspects that are relevant for the practical employment of the dried blood spots method in clinical practice, especially compared to the conventional sampling techniques. Modern chromatographic and electrophoretic techniques for the determination of antidepressants and their metabolites in biofluids were also reviewed [11]. The advantages include their rapidness, high sensitivity, specificity, and miniaturization of liquid chromatography; gas chromatography and capillary electrophoretic methods for the analysis of antidepressants and their metabolites in biofluids were compared. HPLC analysis of β-blockers in biological samples was described by Saleem et al. [12]. Taylor et al. discussed the role of LC-MS/MS in TDM of immunosuppressant and antiretroviral drugs [13].

Sometimes, review articles on TDM of different groups of drugs were published. For example, in 2012, the application of LC-MS/MS for TDM of antifungal, antiviral, immunosuppressant, anticonvulsants, antidepressants, antibiotics, anticancer drugs, and drugs affecting the cardiovascular system was described [4].

This article aims at reviewing the application of liquid chromatography with various detection techniques in TDM. It presents selected examples of liquid chromatography applications and gives some insights on how TDM is benefiting from the tremendous development of liquid chromatography techniques. In this article, we described the application of different chromatographic techniques in combination with the various types of detection used for TDM of drugs belonging to more than one drug classes. We compared the use of different chromatographic systems and detection methods with respect to their selectivity, sensitivity, and specificity.

## 2. Immunosuppressive Drugs

The transplantation of an organ is always followed by a lifelong immunosuppressive therapy to guarantee the survival of the organ in the recipient. Immunosuppressive drugs have to be applied in order to preserve the graft [14]. These drugs are strongly recommended for TDM in order to adjust the adequate dose for each patient to avoid rejection or adverse effects of the therapy [9]. Currently, TDM of immunosuppressive drugs is one of the best established fields of application of TDM. Immunosuppressive schemes in transplantation often include combinations of corticosteroids, calcineurin inhibitors, anti-proliferative agents, and antibody-based therapies. Immunosuppressive drugs have a narrow therapeutic index and show the desired therapeutic effect with acceptable tolerability only within a narrow range of blood concentrations. At low blood levels, there is a risk of organ rejection, while at high blood levels, serious side effects can emerge such as nephrotoxicity, cardiotoxicity, neurological effects, and elevated risk of infections [15]. Additionally, therapeutic ranges of concentration of the different immunosuppressants are also dependent on the transplanted organ, the period after transplantation, the age of the patient, and the comedication. Modern immunosuppressive therapies consist of the combination of at least two immunosuppressants, each of which has different targets, to achieve a lower dose for each drug. Therefore, improving the analytical methods for the TDM of these drugs is very important. Chromatographic methods may be more accurate than immunoassays because of a lower chance of interference from metabolites or matrix. Increasingly, LC-MS/MS is applied for the monitoring of these drugs.

The immunosuppressant drugs that are routinely monitored consist of three main classes: the calcineurin inhibitors ciclosporin and tacrolimus, the mammalian target of rapamycin inhibitors sirolimus and everolimus, and the inosine monophosphate dehydrogenase inhibitor mycophenolic acid.

TDM of immunosuppressive drugs with a narrow therapeutic index is an increasingly popular tool for minimizing drug toxicity while maximizing the prevention of graft loss and organ rejection. Whole blood or plasma samples obtained through venipuncture were used in most procedures for the determination of immunosuppressive drugs. More hydrophobic drugs such as cyclosporine easily penetrate inside red cells, depending on the drug concentration, hematocrit, plasma lipoprotein level, and temperature [16]. Therefore, whole blood is the recommended matrix for this drug concentration monitoring. In many procedures, simple sample preparation was applied involving hemolysis with zinc sulfate and protein precipitation with most often acetonitrile. Automation of the sample preparation step prior to chromatographic analysis is increasingly applied for the pretreatment of biological samples containing immunosuppressive drugs. The separation of immunosuppression drugs is usually carried out on an octadecyl (C18) column using mobile phases containing methanol, rarely acetonitrile, water, formic acid, and ammonium acetate or ammonium formate. The application of two-dimensional (2-D) chromatography is interesting, which is performed on two columns with different separation mechanisms. For the detection of analytes, triple quadrupole MS with an ESI source operated in positive ionization mode was used in most of the proposed procedures. The main important features of analytical methodology for TDM of immunosuppressive drugs are the shortened analysis time and higher sensitivity, selectivity, and specificity. 

### 2.1. HPLC-UV

Since a diode array detector (DAD) can collect an entire spectrum at each time point in a chromatogram, the data are information rich and more selective than single wavelength chromatograms. Ultraviolet spectra together with retention data are applied to identify unknown or suspected drugs and metabolites in various biological samples. The HPLC coupled with diode array detection (HPLC-DAD) is a sufficiently sensitive technique especially for samples that were purified and concentrated before chromatographic analysis [17]. HPLC-DAD is a method that can be also used for screening biological samples in conjunction with a library search algorithm to quickly identify those samples that require confirmatory testing. HPLC-DAD offers many advantages in terms of specificity, sensitivity, speed, and ruggedness. The data produced, comprising both the retention behavior and absorption spectra of eluting chemical entities, result in an identification power at low cost and with widened availability through many laboratories.

Vosough and Tehrani applied HPLC-DAD for the quantification of tacrolimus, everolimus, and cyclosporine in whole blood samples [18]. The samples were simply prepared by the addition of aqueous zinc sulfate solution (0.1 M) and methanol. The mixture was vortexed and then centrifuged. The supernatant layer was evaporated to dryness under a stream of nitrogen. Next, the residues were dissolved in mobile phase in an ultrasonic bath and filtered through a polytetrafluoroethylene (PTFE) syringe filter. After this procedure, samples were injected into an HPLC system. A C18 column and a mobile phase containing acetonitrile and phosphate buffer at pH 3.5 were applied for the separation of analytes. A DAD detector was set to record the 210–400 nm range. Limits of detection (LODs) obtained by the extraction and chromatographic procedures were 0.56, 0.08, and 7.6 μg L^−1^ for tacrolimus, everolimus, and cyclosporine, respectively. Limits of quantification (LOQs) equal to 1.7, 0.24, and 23 μg L^−1^ for tacrolimus, everolimus, and cyclosporine, respectively were received. The proposed procedure is characterized by its simplicity, reducing the organic solvents consumption and short time of analysis [18].

### 2.2. LC-MS

Liquid chromatography in combination with MS detection is the method of choice for the determination of immunosuppressive drugs in biological samples. A single MS lacks sensitivity and specificity compared to a tandem mass spectrometry. The application of advanced tandem and hybrid LC-MS instruments in the field TDM has enabled the determination of lowest concentrations of immunosuppressive drugs even in less purified biological samples. Impressive improvements in detection limits for immunosuppressive drugs, mostly brought about by advances in hyphenated LC-MS techniques, have improved determined concentrations from the microgram to the nanogram per mililiter range [19,20]. The immunosuppressive drugs are often used in combined regimens; in these cases, LC-ESI-MS is especially the best option for the simultaneous analysis of several compounds in one short analysis [21]. Rarely, procedures for the determination of single immunosuppressive drugs were described.

**Figure 1:**
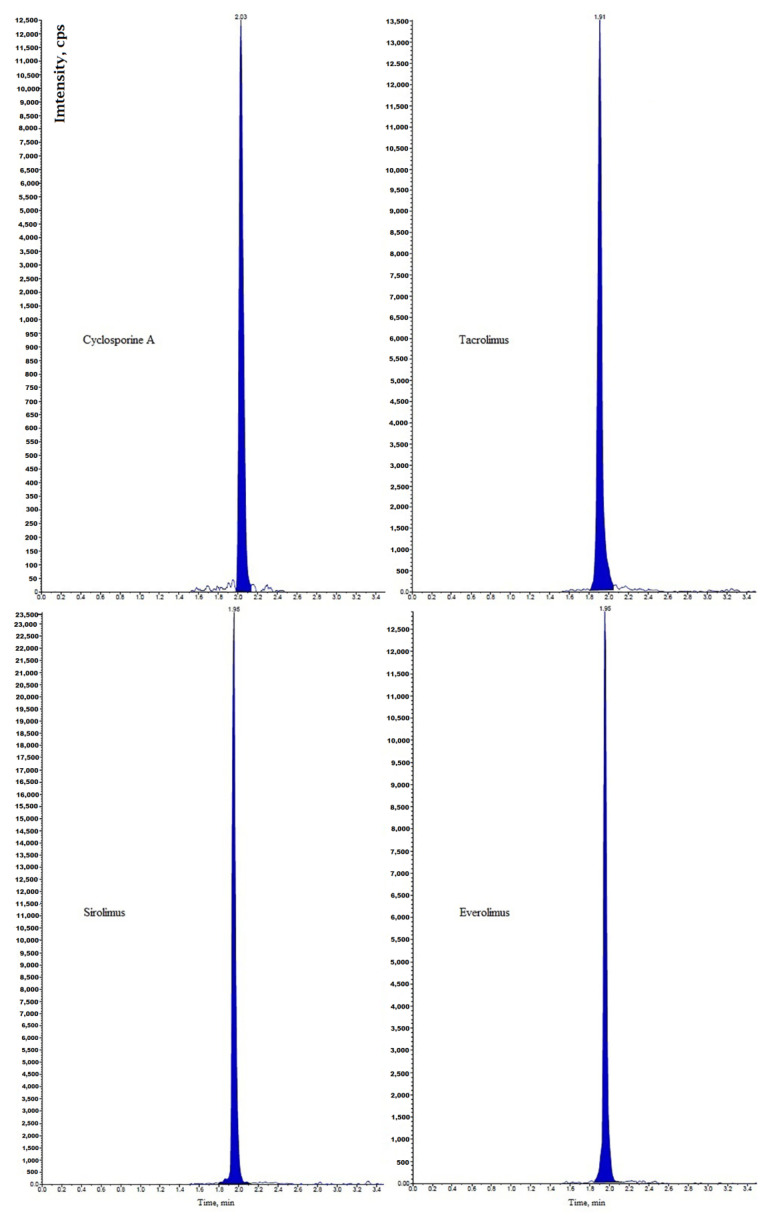
Multiple reaction monitoring (MRM) chromatograms of the immunosuppressants: cyclosporine A (CsA), Tacrolimus (TcR), Sirolimus (SiR), and Everolimus (EvE) in the whole blood of four patients taking different immunosuppressive drugs. The measured concentration was 42.2 for CsA, 6.5 for TrC, 11.1 for SiR, and 7.0 ng mL^−1^ for EvE [15].

Immunosuppressants cyclosporine A, tacrolimus, sirolimus, and everolimus were simultaneously determined in whole blood by LC-MS/MS [15]. Chromatographic separation was performed on a Phenyl-Hexyl column with a mobile phase containing methanol, water, formic acid, and ammonium formate. Multiple reaction monitoring (MRM) chromatograms obtained for investigated immunosuppressants in the whole blood of four patients taking different immunosuppressive drugs is presented in Figure 1. Before analysis, simple samples were prepared by the addition of methanol and 0.5 mol/L ZnSO_4_. The lower limits of quantification (LLOQs) obtained by the described method were set at 0.5 μg/L for tacrolimus, sirolimus, and everolimus and 5 μg/L for cyclosporine A.

Automation of the often time-consuming sample preparation process prior to chromatographic analysis was increasingly used for the pretreatment of biological samples containing immunosuppressive drugs. In particular, the automation of immunosuppressive drugs analysis in whole blood samples is especially difficult because the sedimentation of blood cells occurs within minutes. Marinova et al. developed fully automated protein precipitation-based whole blood samples preparation protocol for quantifying immunosuppressant drugs—sirolimus, everolimus, and tacrolimus—by LC-MS/MS [22]. Samples were prepared by the addition of 0.1 mol/L zinc sulfate aqueous solution into 96-well Multi-Screen Solvinert Filter Plates with a pore size of 0.45 μm low-binding hydrophilic PTFE membrane. Next, internal standard (IS) solution containing ascomycin in a mixture of methanol and water was transferred into the plates. Then, whole blood samples after re-suspension by four times aspiration/dispensation were added, and plates were shaken. Afterwards, protein precipitation by the addition of methanol was performed, and plates were shaken again. Then, the deproteinized supernatants are submitted for on-line solid-phase extraction (SPE), using column switching prior to LC-MS/MS analyses. The investigated immunosuppressant drugs were separated on a C18 column with a mobile phase containing methanol, water, ammonium formate, and formic acid. MS detection was carried out using a triple quadrupole with an electrospray ionization (ESI) source operated in positive ionization mode. For all analytes, the LOD and LLOQ were established at 0.1 μg/L and 0.2 μg/L.

Said et al. developed an on-line microextraction by packed sorbent (MEPS) connected with an LC-MS/MS method for the quantification of cyclosporine, everolimus, sirolimus, and tacrolimus in whole blood [23]. Patient blood samples were diluted with IS working solution and then were centrifuged. Next, the prepared extract was subjected to on-line MEPS preparation. Sample loading was performed by taking six replicates of the diluted blood sample. This was done by withdrawing and ejecting six times into the syringe by the autosampler. Afterwards, the MEPS sorbent was washed with a mixture of methanol and water. The analytes were eluted and injected by withdrawing with a mixture of methanol, isopropanol, acetonitrile, and water. Prepared samples were injected directly into the chromatographic system. The separation of investigated drugs was achieved on a C18 column with a mobile phase containing methanol, water, formic acid, and ammonium formate. Analytes were detected using triple quadrupole MS with ESI operated in positive ionization mode. The LOD obtained by the described procedure was equal to 0.9 ng/mL for cyclosporine and 0.15 ng/mL for everolimus, sirolimus, and tacrolimus. The LLOQ was found to be 3.0 ng/mL for cyclosporine and 0.5 ng/mL for everolimus, sirolimus, and tacrolimus. The advantage of the proposed procedure is the ability to determine analytes in a small sample volume (50 µL), automatization of the sample preparation process, and short time of analysis.

Two-dimensional (2-D) chromatography, based on two independent columns with different separation mechanisms, have proven to be more powerful than one-dimension techniques and have been used successfully to separate and analyze drugs in biological samples with excellent performance [24]. In two-dimensional liquid column chromatography (2D-LC) systems, analytical fractions from the first-dimension column are transferred continuously into the second separation dimension in a sequential and repetitive manner by an in-line transfer valve, while the first-dimension separation continues simultaneously without interruption. Combining different separation modes allows us to optimize the selectivity for a given property distribution in each dimension separately. This results in a huge increase in resolution that cannot be achieved otherwise. The different application modes of liquid chromatography facilitate the separation of complex samples selectively with respect to different analyte properties. The peak capacity in two-dimensional separation is significantly higher due to the fact that each dimension contributes to the total peak capacity as a factor and not as an additive term for single-dimension methods. 

Other examples of liquid chromatography application for TDM of immunosuppressive drugs are presented in Table 1.

## 3. Anticancer Drugs

Cancer is one of the leading causes of death throughout the world. A new era of cancer therapy has emerged in the past decade, with oral targeted anticancer drugs directed against cancer-specific molecules and signaling pathways. Nevertheless, drug resistance, the persistence of cancer stem cells, and adverse drug effects still limit their ability to stabilize or cure malignant diseases in the long term. The main treatments for cancer involve surgery; pharmacological therapy, including chemotherapy; and/or radiation therapy. Cytotoxic drugs are used in the treatment of cancer. The anticancer drugs are classified into several categories: antimetabolites, DNA-interacting agents, molecular-targeting drugs, anti-tubulin agents, and monoclonal antibodies [32]. These drugs are dangerous to handle as they damage normal tissue as well as cancer cells. There are several serious disadvantages associated with the use of anticancer drugs. For example, bone marrow suppression, hair loss, gastrointestinal lesions, nausea/vomiting, and the development of resistance are the common side effects, which are due to non-selectivity, a narrow therapeutic window, and the formation of inactive metabolites. Inter-individual pharmacokinetic variability for treatment by anticancer drugs is often substantial. Sometimes, there may be unsuspected drug–drug interactions due to overlapping drug metabolism or transporter pathways leading. Additionally, standard dosage regimens rarely result in comparable circulating concentrations of the active drug in all patients, possibly favoring the selection of resistant cellular clones or the development of undesirable toxicity [33]. In this respect, the vast majority of anticancer drugs are characterized by a wide spread of plasma concentrations observed following standard dosage regimens, with inter-individual variability. As a consequence, a variation in drug plasma concentration may be observed in some patients, leading to overdrug or underdrug exposures [34]. Thus, the possibility of achieving effective drug concentrations remains a key issue in anticancer chemotherapy. Therefore, the application of TDM is important for mitigating the safety risks and/or minimizing adverse reactions, especially when several of these drugs are used in combination therapy. The instrumental approaches developed in the last decades have permitted the implementation of high-sensitive and specific methods. Various chromatographic methods including HPLC-DAD, HPLC-FLD, and especially LC-MS/MS have been developed for the quantification of many of these drugs in different biological samples.

TDM for individualized anticancer drug dosing by focusing on balancing the therapeutic efficacy and the avoidance of drug toxicity is increasingly applied. Predominantly, anticancer drugs were quantified in plasma samples. In some described procedures, anticancer drug determinations were performed in serum samples. Various sample preparation procedures including protein precipitation, liquid–liquid extraction (LLE), and SPE were utilized. Samples analyzed by HPLC-DAD or HPLC-FLD were most often prepared by protein precipitation followed by SPE or LLE procedures. Biological samples analyzed by LC-MS were often prepared only by simple protein precipitation or rarely by LLE procedures. In some procedures, for sample preparation, SPE was applied usually after protein precipitation. Anticancer drugs were almost always separated on C18 columns. Rarely, another kind of stationary phase was applied. For example, tyrosine kinase inhibitors dasatinib and imatinib were determined in plasma or serum samples by ion exchange chromatography using a strong cation exchange (SCX) column and rituximab was analyzed on a C3 column. Most of anticancer drugs are weak bases for this reason for elution of these drugs mobile phases at acidic pH (containing the addition of acids or acidic buffer) were used in order to suppression of free silanol ionisation. Several chromatographic procedures using mobile phases at basic pH (with the addition of ammonium, amines, or buffers at basic pH) for the suppression of basic analytes ionization were also described. UV detection was relatively often applied for the determination of this group of drugs. Several LC-MS/MS methods have been increasingly developed for the quantification of different anticancer drugs. Fluorescence detection with and without a derivatization process were also utilized for the analysis of some anticancer drugs. TDM of anticancer drugs is still rarely used in clinical practice and requires the development of new, more efficient analytical methods.

### 3.1. HPLC-UV

Anticancer drugs are often administrated in relatively high concentrations; therefore, often, determinations of them are performed by the HPLC-UV method. 

**Figure 2:**
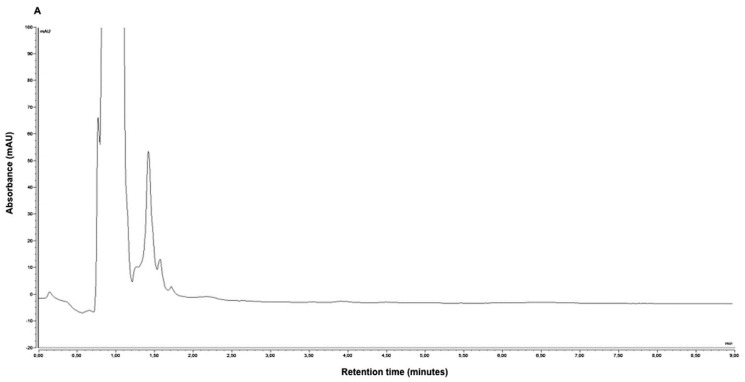

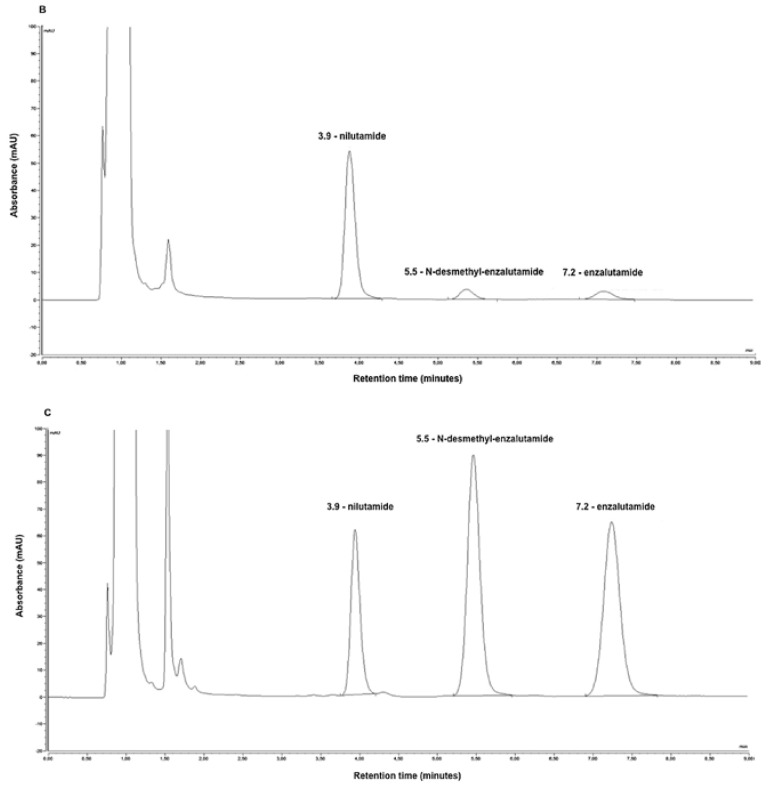
Representative HPLC-UV chromatograms of (**A**) blank human plasma, (**B**) lower limit of quantification of enzalutamide and *N*-desmethyl enzalutamide (0.50 μg/mL for both) and nilutamide (internal standard), (**C**) plasma from a metastatic castration-resistant prostate cancer patient treated with 160 mg of enzalutamide once daily (plasma concentration of enzalutamide and *N*-desmethylenzalutamide: 10.9 μg/mL and 12.4 μg/mL, respectively) [35].

Puszkiel et al. determined enzalutamide, an inhibitor of the androgen-receptor signaling pathway and a competitive inhibitor of dihydrotestosterone, the active metabolite of testosterone, in human plasma samples from patients with metastatic castration-resistant prostate cancer by HPLC-UV [35]. Samples were prepared by the addition of acetonitrile for protein precipitation. Then, samples were vortexed, centrifuged, and evaporated under nitrogen stream. The dry residue was reconstituted in mobile phase, vortexed, ultracentrifuged, and injected into an HPLC system. Chromatographic analysis was carried out on a C18 column. The mobile phase consisted of acetonitrile and ammonium acetate buffer at pH = 4.6. Detection was performed at λ = 270 nm. *N*-desmethyl enzalutamide, the active metabolite of enzalutamide, was also simultaneously quantified by the same procedure. The LOD and LLOQ were 0.20 μg/mL and 0.50 μg/mL for both enzalutamide and its active metabolite *N*-desmethyl enzalutamide, respectively. Representative HPLC-UV chromatograms of (A) blank human plasma, (B) lower limit of quantification of enzalutamide, *N*-desmethyl enzalutamide (0.50 μg/mL for both), nilutamide (internal standard), and (C) plasma from a metastatic castration-resistant prostate cancer patient treated with 160 mg of enzalutamide once daily are presented in Figure 2. The peaks obtained on the chromatogram were symmetrical and well separated. The sample preparation and HPLC-UV analysis procedure was successfully applied to monitor plasma enzalutamide and *N*-desmethylenzalutamide concentrations in 16 patients. 

The introduction of tyrosine kinase inhibitors in the clinical practice revolutionized cancer therapy by converting some previously deadly malignancies into chronic diseases. The tyrosine kinase inhibitors are also applied for the treatment of other conditions, such as autoimmune diseases. After the oral drugs administration, complex steps in drug absorption and metabolism are responsible for the large inter- and intra-individual variability of tyrosine kinase inhibitors’ plasma levels. Therefore, due to the high variability of tyrosine kinase inhibitors levels and their therapeutic and toxic effects, the concentration of the drugs in patients should be monitored. For the determination of ponatinib, a third-generation tyrosine kinase inhibitor used for optimally inhibiting native and mutated BCR-ABL, including the gatekeeper mutant T315I, the HPLC-UV method was developed [36]. Plasma samples were prepared by an SPE procedure performed on Oasis HLB extraction cartridges. Cartridges were activated previously with methanol and then water, which was followed by washing with water and then 60% methanol in water. Elution was performed with methanol. Eluates were dried by vortex–vacuum evaporation using a rotary evaporator. Dry residues were reconstituted with methanol and vortexed. Then, a mobile phase was continuously added to each sample. Next, samples were vortexed for and injected into the HPLC system. Plasma samples were analyzed on a C18 MGII column with the sealing of silanol by an application of ultimate polymer coating, ensuring a good peak profile even under the neutral condition, which is the most severe for the peak profile of basic compounds. For the elution of investigated drugs, a mobile phase containing acetonitrile, water, and potassium dihydrogen phosphate (pH = 3.5) was applied. The detection of analytes was carried out at a wavelength of 250 nm. The authors declare that the accuracy and precision of the proposed HPLC-UV method for the quantification of ponatinib is similar to those of the previously described LC-MS/MS methods, and the sensitivity was slightly superior. The LOD and LOQ values obtained for ponatinib by the procedure were 0.5 and 1.0 ng/mL, respectively.

### 3.2. HPLC-FLD

Fluorescence detection can provide distinct advantages in sensitivity and selectivity over absorbance detection, and it can be useful for the analysis of various drugs in biological samples. The FLD detector in some cases allows for almost 30 times higher sensitivity than the UV detector, which is particularly important for the examination of biological samples, where analytes concentrations are low. Sometimes, a fluorescence detector was applied for the determination of anticancer drugs without or with derivatization.

**Figure 3:**
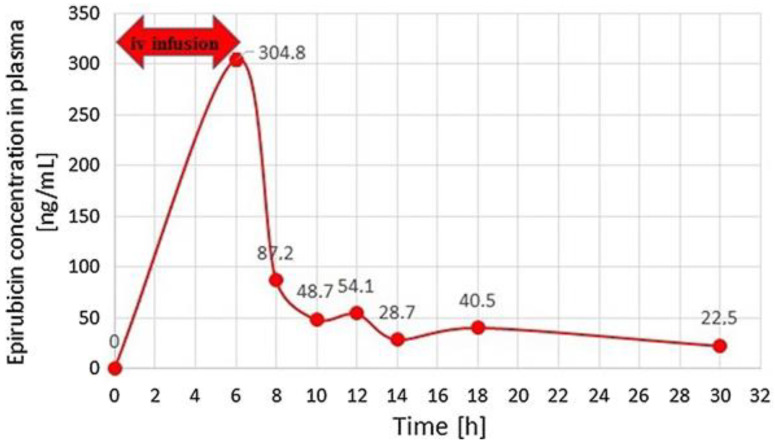
The profiles of epirubicin concentrations in plasma samples from a 19-year-old patient with metastatic alveolar rhabdomyosarcoma after a 6-hour intravenous infusion of epirubicin (150 mg/m^2^) [37].

Treder et al. developed and validated the high-performance liquid chromatography with fluorescence detection (HPLC-FLD) method for the quantitation of epirubicin, which is a drug acting by intercalating DNA strands in human urine and plasma [37]. For the preparation of urine and plasma samples, SPE followed by protein precipitation with different deproteinizing agents was applied. After the addition of IS, samples were vortex-mixed and then ZnSO_4_ in water to urine samples and 0.1 M HCl to plasma samples were added. Afterwards, the prepared solutions were mixed and shaken mechanically. Next, after centrifugation, the samples were extracted on Supel Select HLB SPE cartridges. The cartridges were previously activated by running water and methanol. Then, the samples were introduced to the SPE cartridges, washed with water, and dried. For the elution of epirubicin and the internal standard, a mixture containing dichloromethane, 2-propanol, and methanol was used. The samples were evaporated to dryness under reduced pressure, and the dry residue was reconstituted in a mixture of acetonitrile and water. The HPLC-FLD analysis was carried out on a Synergi Hydro-RP column with a mobile phase consisting of acetonitrile and phosphate buffer at pH 4.1. Epirubicin was monitored at 497 nm for excitation wavelengths and 557 nm for emission wavelengths. Due to naturally occurring epirubicin fluorescence, the derivatization reactions were not necessary in the procedure. Low values of LOD (0.25 ng/mL) and LOQ (0.5 ng/mL) were obtained for both plasma and urine samples. The methodology was successfully applied for the monitoring of epirubicin concentrations in plasma and urine samples from patients. The profiles of epirubicin concentrations measured by the described method in plasma samples from a patient with metastatic alveolar rhabdomyosarcoma after a 6-hour intravenous infusion of epirubicin (150 mg/m^2^) are presented in Figure 3.

For the determination of analytes that neither contain any strong chromophores nor fluorophores in their molecule by HPLC-UV or HPLC-FLD, a suitable derivatization method is necessary. L-Asparaginase, applied to the treatment of childhood acute lymphoblastic leukemia, was determined in patients’ serum by HPLC-FLD after derivatization reaction [38]. For sample preparation, serum was deproteinized with 4% sulfosalicylic acid solution. After vortexing and centrifugation, IS was added. The solution was diluted by mobile phase and alkalized with NaOH (phenolphthalein solution was used as the indicator). Then, the precolumn derivatization was performed with o-phthalaldehyde–sulfhydryl reagent and next, samples were injected into the chromatographic system. Chromatographic analysis was performed on an AAA (Amino Acid Analysis) column using a mobile phase composed of acetonitrile, methanol, and sodium acetate (pH 7.2). A fluorescence detector operating at an excitation wavelength of 340 nm and emission wavelength of 444 nm was applied for the detection of analytes. The LLOQ was determined at 0.76 nmol/mL. 

### 3.3. LC-MS

For the determination of anticancer drugs in biological samples, the LC-MS/MS method using ESI as an ion source as well as a triple quadrupole analyzer are often reported. For samples preparation, a simple protein precipitation was often used.

Koller et al. developed an LC-MS/MS method for the simultaneous monitoring of 11 tyrosine kinase inhibitors (imatinib, dasatinib, nilotinib, bosutinib, ponatinib, ruxolitinib, brutinib, filgotinib, tofacitinib, baricitinib, and peficitinib) in human plasma [39]. Endogenous blood plasma phospholipids, proteins, salts, other blood plasma components, and exogenous substances can influence the chromatographic separation of analytes and the ionization process. Therefore, the application of an appropriate sample sample preparation method is very important. For sample preparation, the authors tested SPE and simple protein precipitation methods. For protein precipitation, acetonitrile with 0.1% formic acid was applied. Next, the mixture was centrifuged and vaporated. Finally, the dry residue was reconstituted with 5% NH_4_OH in methanol/water mixture. SPE was performed by the application of a mixed-mode cation exchange sorbent (PRiME μ-SPE MCX) containing both reversed-phase hydrophilic-lipophilic sorbent and ion-exchange functionality for orthogonal sample preparation. The human plasma samples were loaded into the Oasis PRiME μ-SPE MCX 96-well μElution Plate. After the loading step, the samples were washed by a mixture of ammonium formate and formic acid in water, which was followed by washing by methanol. Next, the analytes were eluted with a mixture of NH_4_OH, methanol, and water. The eluate was injected into the LC-MS/MS system. The use of μ-SPE allowed for endogenous phospholipid elimination and resulted in high extraction recoveries, no significant matrix effect, and exceptional overall process efficiency. Tyrosine kinase inhibitor drugs were separated on a C18 column with a mobile phase containing acetonitrile, water, and ammonium hydroxide. For the detection of analytes, triple quadrupole MS equipped with ESI was applied. The quantification of investigated drugs in the method was performed in the positive and dynamic multiple reaction monitoring (dMRM) mode.

**Figure 4:**
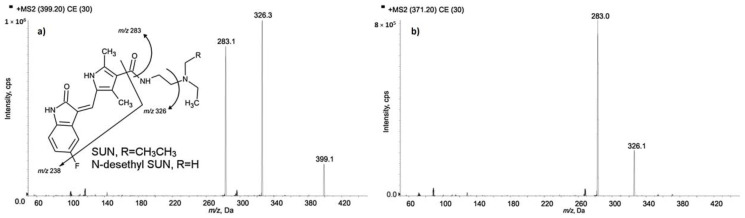
MS/MS mass spectra of sunitinib (SUN) (**a**) and *N*-desethyl SUN (**b**) with chemical structures and identification of the main fragment ions (CE = 30 V); the 238 m/z fragment was obtained with CE = 60 V for both the analytes [40].

Sunitinib is used in therapy of advanced renal cell cancer, imatinib-resistant or -intolerant gastrointestinal stromal tumors, and pancreatic neuroendocrine cancers. Plasma exposure of the drug shows large inter-individual variations. The narrow therapeutic window and the positive ratio of exposure to the effectiveness of sunitinib indicates the need for its therapeutic drug monitoring. For the determination of sunitinib and its active metabolite *N*-desethyl sunitinib in patients’ plasma samples, the LC-MS/MS method was applied [40]. In solution, sunitinib and *N*-desethyl sunitinib undergo photoisomerization and procedures of samples collection and handling under strict light protection. The authors proposed a method based on a simple and fast procedure that quantitatively reconverts the E-isomer of both analytes, obtained during sample draw and processing without light protection, into their Z-forms. Before analysis, samples were vortex-mixed and centrifuged. An aliquot of sample was transferred to an Eppendorf polypropylene tube, IS was added, and the mixture was vortex-mixed. Then, protein precipitation was performed by the addition of methanol. In the next step of sample preparation, a supernatant was transferred to a borosilicate glass vial with a pre-slit PTFE cap. The vial containing the supernatant was heated at 70 °C in a thermostatic bath to revert the isomerization and thus to obtain only the active Z-isomer of the drug. Then, the samples were transferred to the autosampler in the dark. The separation was performed on a C18 column with a mixture of acetonitrile, water, and formic acid as a mobile phase. For the detection of analytes, an HPLC system was coupled to a triple quadrupole MS operated in positive ionization mode. The LODs obtained by the proposed method were 12 pg/mL for sunitinib and 15 pg/mL for *N*-desethyl sunitinib. The LLOQ values were established on the basis of the concentration range forecast in patients’ samples and fixed at 0.1 ng/mL for both analytes. One of the advantage of the method, especially regarding its use in TDM, is the small plasma sample volume of only 30 µL. MS/MS mass spectra of sunitinib and (a) *N*-desethyl sunitinib (b) with chemical structures and identification of the main fragment ions (CE = 30 V), as well as the 238 m/z fragment obtained with CE = 60 V for both the analytes, are presented in Figure 4.

### 3.4. HPLC-UV and LC-MS

Some authors proposed both HPLC-UV and LC-MS procedures for the determination of anticancer drugs in biological samples. 

Methotrexate, a folate antagonist that acts by inhibiting dihydrofolate reductase to prevent cancer cell division, was determined by HPLC-UV and LC-MS/MS in human plasma samples [41]. For LC-UV, samples were prepared by the SPE procedure performed on Strata Screen-C cartridges; for LC-MS/MS, samples were prepared by protein precipitation with acetonitrile. Methotrexate was analyzed by LC-UV on a C18 column with a mobile phase containing methanol, water acetic acid, and triethylamine. The UV detection wavelength was set at 306 nm. Analysis of the drug by LC-MS/MS was performed on a C18 column using a mixture of acetonitrile, water, and acetic acid as a mobile phase. In LC-MS/MS, detection was performed on triple quadrupole MS with an ESI source operated in positive mode. An LLOQ = 0.05 μmol/L was obtained by both LC-UV and LC-MS/MS. The authors compared results obtained by LC-MS/MS with HPLC-UV by measuring 42 clinical samples. The statistical results showed that the two assays were in good agreement. Both analytical methods resulted in accurate and sensitive outcomes.

Most analyses of anticancer drugs are carried out on columns with alkylbonded stationary phases—most often C18, rarely C8. There are only a few reports on the analysis of anticancer drugs performed on other types of columns, e.g., strong cation exchange (SCX). HPLC-UV and LC-MS/MS methods were developed for the tyrosine kinase inhibitors dasatinib and imatinib analysis in plasma or serum samples [42]. The sample volumes used for drug determination were 100 µL and 50 µL for HPLC-UV and LC-MS/MS, respectively. Before chromatographic analysis, IS solution and Tris solution (2 mol/L, pH 10.6) were added to samples. After vortex-mixing, butyl acetate and butanol were added. Then, vortex-mixing and centrifugation were performed. Next, the upper layer was transferred into a chromatographic system. In both chromatographic methods, the separation of analytes was carried out on an SCX column. For an LC-UV mobile phase containing methanol, ammonium perchlorate and sodium hydroxide (pH = 7.5) were applied. For LC-MS/MS, investigated drugs were analyzed with using a mobile phase containing methanol and ammonium acetate adjusted by perchloric acid to pH 6.0. A UV-Vis detector was set at 270 nm. The detection of analytes in LC-MS/MS was performed on triple quadrupole MS with atmospheric pressure chemical ionization (APCI) operated in positive mode. LODs obtained by HPLC-UV were set at 0.001 mg/L for imatinib and 0.005 mg/L for norimatinib. LLOQ values were 0.002 mg/L and 0.01 mg/L for imatinib and norimatinib, respectively. LODs obtained by LC-MS/MS were 0.002 mg/L for imatinib, 0.001 mg/L for norimatinib, and 0.08 mg/L for dasatinib; LOQs were 0.003 mg/L for imatinib, 0.002 mg/l for norimatinib, and 0.13 mg/L for dasatinib. The proposed LC-MS/MS approach was compared with the HPLC method by measuring 42 clinical samples. The authors concluded that the statistical results showed that the two methods were in good agreement. 

Additional examples of the application of liquid chromatography for determining anticancer drugs concentration in biological samples are presented in Table 2.

## 4. Antibiotics 

Antibiotics are widely prescribed drugs, but problems with organisms developing resistance to these drugs means that their efficacy may be lost and care should be taken to avoid unnecessary prescription. Optimizing the prescription of antibiotics is required to improve clinical outcome from infections and to reduce the development of antimicrobial resistance. An adequate antibiotic treatment regime is a crucial factor for especially the outcome of patients suffering from serious infections, respectively sepsis. The pharmacological effect of antibiotics on the critically ill is difficult to study without first considering the changes in physiological antimicrobial agents, which is an essential step in the treatment of infections. Different classes of antibiotics work in different ways to treat bacterial infections. Some antibiotics, e.g., the penicillins, have a wide therapeutic index; therefore, the measurement of plasma levels is only rarely necessary. Other classes, e.g., the aminoglycosides, have dose-related toxic effects, and therapeutic drug monitoring is often needed. An appropriate antibiotic treatment regime is a crucial factor for the result of patients suffering from serious infections. TDM is a very useful tool to optimize antibiotic therapy. TDM of antibiotics is used to personalize dosing to achieve antimicrobial exposures associated with a high probability of therapeutic success and suitably low probabilities of toxicity and generation of antimicrobial resistance [56]. Currently, clinicians are increasingly employing TDM of various antibiotics, especially beta-lactam antibiotics to ensure adequate antibiotic exposure. Measuring biological sample concentrations of antibiotics provides the possibility of dose adjustments to optimize the treatment of patients. Several chromatographic methods have been described for the determination of antibiotics in plasma or serum and rarely other biological samples.

Most often, the concentration of antibiotics was measured in plasma samples, rarely in serum. Sometimes, analytical procedures for the determination of antibiotics in other samples, e.g., saliva, bile, or bronchoalveolar lavage were described. Protein precipitation and/or LLE extraction was frequently used for sample preparation, especially when samples were next analyzed by LC-MS. In several procedures, the SPE was applied for sample preparation particularly before analyses were carried out by HPLC-DAD or HPLC-FLD. For separation, C18 columns were commonly applied. There are only a few procedures where antibiotics were separated on columns with a phenyl stationary phase. In most cases, mobile phases applied for antibiotics analysis contained an organic modifier, water, and various acids or acidic buffers.

Examples of procedures used for the determination of antibiotics in various biological samples using liquid chromatography with different detection methods are presented below.

### 4.1. HPLC-UV

HPLC in combination with UV detection is the method quite often applied for the determination of most antibiotics in biological samples. The method is often suitable for the determination of antibiotics because they are frequently used in high doses, and the concentration achieved in blood or other tissues is so high that their quantification by the HPLC-UV method is possible.

The HPLC-UV method for therapeutic drug monitoring of piperacillin and tazobactam was described by Verhoven et al. [57]. A chromatographic analysis of blood samples was performed on a C18 column with a mobile phase containing methanol, acetonitrile, and trifluoroacetic acid. The analyte peaks were detected by UV absorbance at 214 nm. The authors noted that in the critically ill patient population, the protein binding of piperacillin is highly variable and ought to be considered carefully in any estimation of the antibiotic concentration. Therefore, measuring free piperacillin concentrations instead of total drug concentrations is more advisable. 

Imipenem, a beta-lactam antibiotic, is rapidly distributed to most tissues and renally cleared. Patients with severe infections are known to manifest pathophysiologic phenomena, such as augmented renal clearance and increased volumes of distribution, and they may have subtherapeutic plasma concentrations [58]. For this reason, TDM is advisable for patients treated with imipenem. Bricheux et al. analyzed imipenem concentrations in patients’ plasma samples by HPLC-DAD. Imipenem was analyzed on a C18 column and detected at 298 nm. The LLOD and LLOQ obtained by the method were 0.2 mg/L and 0.5 mg/L, respectively.

**Figure 5:**
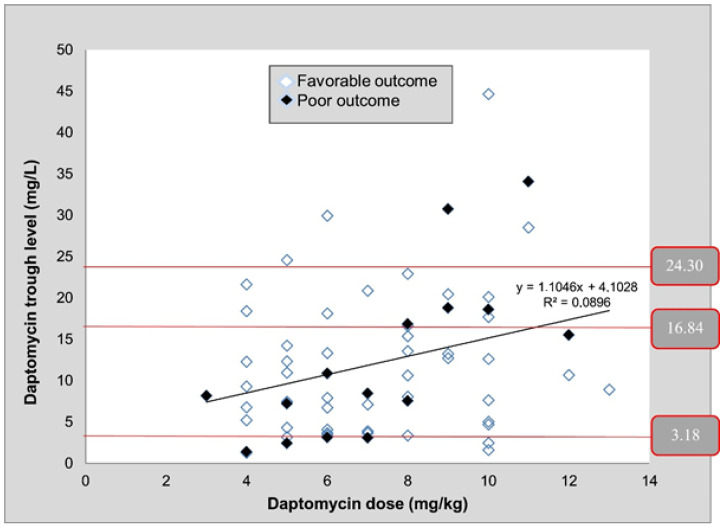
Correlation of daptomycin dose through serum level (C min) and clinical outcome [59].

HPLC with a UV/Vis absorbance detector was used for the determination of daptomycin, a cyclic lipopeptide antibiotic with potent bactericidal activity against most Gram-positive organisms including vancomycin-resistant enterococci, methicillin-resistant staphylococci, and ‘heterodrug-resistant’ glycopeptide-resistant *Staphylococcus aureus* [59]. The antibiotic was quantified in blood samples collected after 3 or more days post-treatment initiation. A chromatographic analysis of daptomycin was carried out on a C18 column with a mixture of acetonitrile, water, and ammonium phosphate as a mobile phase. Detection was performed at λ = 220 nm. A high variability in daptomycin use and patent’s serum levels was detected. Figure 5 shows how C minimum values varied widely despite the administration of 4, 6, or 8 mg/kg of daptomycin and the correlation with clinical outcome. It confirms that TDM might be useful to optimize daptomycin doses and to avoid therapeutic failure.

Daptomycin was also determined by HPLC-UV in plasma samples [60]. Before chromatographic analysis, samples were extracted in different conditions. The highest recovery of daptomycin was obtained when LLE extraction was performed with a mixture of acetonitrile and 85% H_3_PO_4_ in water. A C18 column and mobile phase containing acetonitrile and phosphate buffer at pH 3.2 were used for chromatographic analysis. The LOD and LOQ values obtained in the investigations were 1.65 and 5.00 mg/L, respectively. Then, the method was applied to measure daptomycin concentration in 122 plasma samples from patients treated with the drug. The proposed HPLC-UV method was compared with the commercially available LC-MS/MS method. Correlation between results obtained by both methods for 122 plasma samples was high (r = 9474).

Antibiotics most often were determined in plasma, serum, or whole blood, but sometimes, they were also determined in other biological samples. 

Linezolid, an oxazolidinone derivative with a bacteriostatic effect against Gram-positive bacteria, including methicillin-resistant *Staphylococcus aureus*, vancomycin-resistant enterococci, and cephalosporin-resistant *Streptococcus pneumonia* was determined in human plasma and in bronchoalveolar lavage [61]. Samples were prepared by SPE on C18 cartridges, because it provided a quantitative extraction of the antibiotic, with purer and more concentrated samples compared to more often applied liquid–liquid extraction. The SPE cartridges were sequentially pre-conditioned with methanol, acetonitrile, and purified water. After the loading of plasma or bronchoalveolar lavage samples, cartridges were washed with 5% acetonitrile in water and then eluted with methanol. The eluted solutions were evaporated to dryness. The residues were reconstituted in a mobile phase solution and then transferred into a chromatographic system. LC-UV analysis was performed on a C18 column with a mobile phase containing acetonitrile and dihydrogen phosphate buffer (pH 3.5). The UV detection wavelength was set at 254 nm. The LLOQ value was determined as 25 ng/mL for both samples. 

### 4.2. HPLC-FLD

Rarely, the application of HPLC with fluorescence detection for the determination of antibiotics was reported. 

Colistin, the most important drug used for treating infections caused by multidrug-resistant Gram-negative bacteria, was determined by HPLC-FLD with derivatization in human plasma samples [62]. Due to the dose-related side neurotoxicity and nephrotoxicity effects of colistin, monitoring of the drug plasma concentration is important for its dose adjustment. In the procedure for sample preparation, the authors proposed in-SPE derivatization for simultaneous sample cleanup and derivatization. In the first step of the sample preparation, protein precipitation was performed by the addition of methanol and trichloroacetic acid. Then, samples were centrifuged and supernatant was transferred into a new glass tube. Afterwards, NaOH was added, and samples were vortex-mixed. Next, a mixture containing 0.01 M HCl and methanol was added and samples were vortex-mixed. In the next step of sample preparation, an SPE procedure was performed using SPE C18 cartridges. The cartriges were conditioned sequentially with methanol and carbonate buffer at pH 10. Then, the deproteinized plasma samples were loaded into the SPE cartridge. After washing the cartridge with carbonate buffer (pH = 10), the derivatization agent 9-fluorenylmethyl chloroformate was added. After 10 min of reaction, the cartridges were dried under the vacuum manifold. Next, the cartridges were washed with 95% of methanol in water, followed by the elution of reaction products with acetone. The effluent was mixed with 6 M boric acid. The resulting solution was filtered through a cellulose filter and transferred into a chromatographic system. The proposed sample preparation procedure allowed for simultaneous sample cleanup and derivatization in one step. A C18 column and mobile phase containing acetonitrile, tetrahydrofuran, and water were employed for chromatographic separation. A fluorescence detector with an excitation wavelength of 260 nm and an emission wavelength of 315 nm was applied for the detection of derivatized analytes. LOD and LLOQ obtained by the described procedure were set at 0.1 μg/L and 0.3 μg/L, respectively. The proposed HPLC-FLD procedure was applied to quantify the colistin concentrations of plasma samples obtained from two patients in an intensive care unit.

### 4.3. LC-MS

LC-MS/MS has replaced HPLC-UV in many clinical laboratories, especially in high-income countries. The one advantage of LC-MS, because of its sensitivity and selectivity, is that the use extensive sample extraction techniques such as SPE and LLE is not required. Thus, additionally, the time of the analysis is significantly reduced, which is especially salient for TDM of antibiotics. Several LC-MS/MS procedures were developed for the quantitation of antibiotics belonging to various groups.

Teicoplanin, a glycopeptide antibiotic used for the treatment of severe Gram-positive infection, was determined in human serum by LC-MS/MS. For the drug, approximately 10 days, but rarely 14 days is needed to reach a steady state of its concentration in a patient’s organism. The inter-individual variability in teicoplanin concentrations may be due to the characteristics of the population such as age, underlying disease, renal function, and serum albumin. For this, the TDM in treatment with this drug is necessary. Jung et al. developed the LC-MS/MS method for the determination of teicoplanin in human serum [63]. Before LC-MS/MS analysis, serum samples were protein-precipitated by the addition of 10% sulfosalicylic acid dehydrate. After the addition of IS, samples were centrifuged and injected into an HPLC system. Separation was performed on a C18 column with a mobile phase containing acetonitrile, water, and formic acid. The LLOD obtained by the analytical procedure was 0.01 µg/mL and the LLOQ was 2.0 µg/mL. The described procedure was applied to measure teicoplanin concentration in 421 serum samples from patients treated with the antibiotic. The authors detected the high proportion of subtherapeutic teicoplanin concentrations in investigated samples; therefore, TDM of the drug is very important.

Multidrug therapies for tuberculosis treatment are commonly applied in clinical practice to achieve better treatment outcomes. However, drug resistance and adverse reactions come along with these therapies, and TDM is a feasible way to precaution them. TDM of antituberculosis drugs may bring reduced intra and inter-individual variability in their plasma levels and pharmacokinetically guided dose adjustments, as well as a significantly improved efficacy and tolerability. Tuberculosis is often treated with a cocktail of antimicrobial drugs to prevent acquired drug resistance and treatment failure. For this reason, for TDM of antituberculosis drugs, precise analytical methods for the simultaneous determination of a mixture of drugs must be developed. For the simultaneous analysis of these drugs, HPLC-UV was rarely applied, because the method required an optimized liquid-liquid extraction or solid phase extraction often in conjunction with a concentration step. Such extraction and concentration procedures require a relatively high biological sample volume (e.g., 1000 μL). With multiple analytes, the extraction targets become more heterogeneous with potentially broad disparities in chemical properties, such as polarity and molecular weight, making it extremely complex or impossible to achieve high extraction recoveries for all compounds without compromising the sample cleanup itself. Therefore, most antituberculosis drug analysis procedures were developed using LC-MS/MS.

The LC-MS/MS method for the simultaneous determination of pyrazinamide, isoniazid, ethambutol, streptomycin, and rifampicin in human plasma was developed [64]. The samples underwent a simple pretreatment by protein precipitation with methanol containing 1% formic acid. For obtaining a best separation selectivity, optimal retention, and symmetrical peaks of the analytes, the authors tested amide, pentafluorophenyl, and C18 columns and various mobile phases containing methano or acetonitrile and the addition of formic acid, ammonium acetate, or heptafluorobutyric acid. The best results were achieved on a C18 column with a mobile phase containing methanol, water, formic acid, and heptafluorobutyric acid used as an ion-pairing reagent. Triple quadrupole MS with an ESI source operated in positive ionization mode was coupled to a chromatographic instrument for the detection of anlytes. LLOQs obtained by the method were from 0.2 ng/mL for ethambutol to 2 μg/mL for streptomycin. The usefulness of the procedure in clinical practice was confirmed by determining all the analytes in plasma samples obtained from 33 tuberculosis patients. 

Baietto et al. applied a UPLC-MS-MS method for the simultaneous quantification of first-line antituberculars: ethambutol, isoniazid, pyrazinamide, rifampicin, thymidine, and 6,7dimethyl-2,3-di(2-pyridyl)quinoxaline in plasma and peripheral blood mononuclear cells [65]. Plasma samples were prepared by protein precipitation with acetonitrile followed by centrifugation. Next, supernatant was diluted 1:10 with water and injected into the UPLC system. Samples of peripheral blood mononuclear cells were vortexed and then sonicated in a water bath. After sonification, samples were centrifuged and the supernatant was evaporated in a vacuum centrifuge at 60 °C, reconstituted with a mixture of water and acetonitrile, and injected into the UPLC system. The separation of analytes was performed on a UPLC HSS T3 column. LOD values obtained for samples of plasma and peripheral blood mononuclear cells were 58 and 2.93 ng/mL, respectively, for ethambutol, 58 and 0.391 ng/mL, respectively, for isoniazid, 68 and 0.391 ng/mL, respectively, for pyrazinamide, and 117 and 0.976 ng/mL, respectively, for rifampicin. Authors observed high inter-patient variability both in plasma and intracellular concentrations, which confirms the advisability of using TDM for investigated drugs.

Additionally, examples of the application of LC for the analysis of antibiotics are presented in Table 3.

## 5. Antifungal Drugs

Fungal infection is divided into two classes: superficial fungal infections and invasive fungal infections. Superficial fungal infections are limited to the outer layer of the body, e.g., tinea onychomycosis. Invasive fungal infections are also a serious and widespread clinical problem and are one of the most frequent causes of morbidity and mortality especially in high-risk groups such as patients who are on immunosuppressive therapy or cancer chemotherapy. Most invasive fungal infections are found deep inside of the solid organs and/or bloodstream in the immune-deficient population. Some commonly encountered species that cause invasive fungal infections are *Candida* spp., *Aspergillus* spp., and *Cryptococcus* spp. [78]. Invasive fungal infections are more frequently found in people with weakened immune systems. Many patients with hematological or oncological diseases and undergoing hematopoietic stem cell transplantation suffer from invasive fungal infections. These life-threatening conditions result from a patient’s immunodeficiency that is a consequence of immunosuppressive therapy. These patients need very effective antifungal therapy. Curently, patients after transplantations routinely receive antifungal agents for prophylaxis and the curative treatment of fungal infection. As a result of the high toxicity of most antifungal drugs, one needs to ensure that drug exposure at the site of infection is adequate while toxicity to the host is minimized. Many antifungal drugs exhibit great inter- and intra-individual variability in blood concentrations due to variable absorption, metabolism, elimination, non-linear pharmacokinetics, drug–drug interactions, and cytochrome P450 polymorphisms [7]. TDM can be used for tailoring medications to achieve exposures associated with a high probability of therapeutic success, decrease treatment-related toxicity, and help prevent the emergence of antifungal resistance due to subtherapeutic drug exposures.

There are five main classes of antifungal drugs: polyenes, flucytosine, allylamines, azoles, and echinocandins [7]. TDM is especially justified for flucytosine and triazoles due to their large inter- and intra-individual pharmacokinetic variability and their high tendency for drug–drug interactions. The LC-MS/MS is preferred for the determination of antifungal drugs in biological samples due to its superior analytic sensitivity and specificity. 

The concentration of antifungal drugs was measured in most described procedures in plasma or serum samples, but some papers described the application of other biological samples, e.g., saliva. Most often, samples were prepared simply by protein precipitation, which was sometimes followed by LLE and rarely by SPE. Analytes were mainly separated on C18 columns using a mobile phase containing organic modifier, water, and the addition of acids or acidic buffer. The application of mobile phases containing only an organic modifier and water was also often reported. In most procedures developed for antifungal drugs, determinations in biological samples were performed by LC-MS/MS using triple quadrupole MS with an ESI source operated in positive ionization mode. LC-MS/MS exhibits superior sensitivity and specificity with simple sample preparation and a short analytical time.

### 5.1. HPLC-UV

There are a few examples of procedures for analyzing antifungal drugs using UV detection. HPLC-UV does not require the use of complicated equipment, well-trained personnel, and higher costs, which makes it possible to widely use it in clinical laboratories.

Voriconazole, itraconazole, and posaconazole are highly efficient in the treatment of invasive fungal infections. However, therapy using these requires the monitoring of these drugs’ plasma concentrations, due to a narrow therapeutic index and large inter-individual variability of its pharmacokinetics. The HPLC-DAD method for the simultaneous quantification of voriconazole, itraconazole, and posaconazole, as well as some of their major metabolites, voriconazole N-oxide and hydroxy-itraconazole, in human serum is reported [79]. In the procedure, samples were prepared by a simple method of protein precipitation with acetonitrile. After centrifugation, samples were transferred into an HPLC system for analysis. The investigated compounds were separated on a C18 column. A mixture containing only acetonitrile and water was applied as a mobile phase. Analytes detection was performed by monitoring the absorbance at three wavelengths (255, 266, and 311 nm). Their identification was confirmed by comparing retention times and UV spectra against the standards. The LLODs obtained by the procedure were between 0.06 and 0.125 mg/L, while LLOQs were between 0.25 and 0.125 mg/L. The proposed procedure allows having results for clinical decisions in 45 minutes, including the sample preparation and chromatographic analysis.

### 5.2. HPLC-FLD

Very rarely, antifungal drugs were analyzed by HPLC with fluorescence detection. 

Voriconazole was determined in human plasma by HPLC with fluorescence detection [80]. Total and a free fraction of the drug was determined. Before HPLC analysis, plasma samples were prepared by protein precipitation using acetonitrile followed by the free drug isolation from plasma applying ultrafiltration. Chromatographic separation was performed on a C18 column with a mobile phase containing acetonitrile, phosphate buffer at pH 6.5, and *N,N,N′,N*″- tetramethylethylenediamine. The excitation wavelength was 254 nm, while the emission wavelengths were 385 nm (0–6.2 min) and 450 nm (6.2–9.0 min). The LLOQ for the analysis of total voriconazole was 0.1 µg/mL, and it was 0.05 µg/mL for the analysis of the free fraction. The advantages of the described procedure are the possibility of determining the drug in a small volume of plasma samples and a simple and fast extraction procedure. The usefulness of the analytical procedure was confirmed by the determination of voriconazole in clinical samples collected from pediatric patients undergoing antifungal therapy with the drug.

### 5.3. LC-MS

In order to maximize the clinical benefit of TDM, accurate quantitative results are required with minimal turnaround times. LC coupled with tandem MS is the method that allows the selective and sensitive quantification of several drugs in a short time. Most methods for the chromatographic analysis of the antifungal drugs proposed used MS detection. 

Antifungal drugs such as voriconazole, posaconazole, itraconazole, and hydroxy-itraconazole were analyzed in human plasma using LCMS/MS [81]. After protein precipitation, samples were vortexed and centrifuged and then injected into LC-MS/MS. The separation of analytes was carried out on a UPLC BEH C18 column. The mobile phase applied in the procedure contained methanol, water, and ammonium acetate. For analytes detection, triple quadrupole MS operated in positive ESI mode was coupled to an LC system. The LODs achieved by the described procedure for voriconazole, posaconazole, itraconazole, and hydroxyitraconazole were 0.09 mg/L, 0.07 mg/L, 0.02 mg/L, and 0.04 mg/L, respectively. The LLOQ defined by a CV and target deviation of < 20% for all the antifungal triazoles tested was 0.10 mg/L. The following results were obtained: voriconazole CV 4%, bias 13%; posaconazole CV 9%, bias 11%; itraconazole CV 5%, bias 11%; and hydroxy-itraconazole CV 6%, bias 16%.

**Figure 6:**
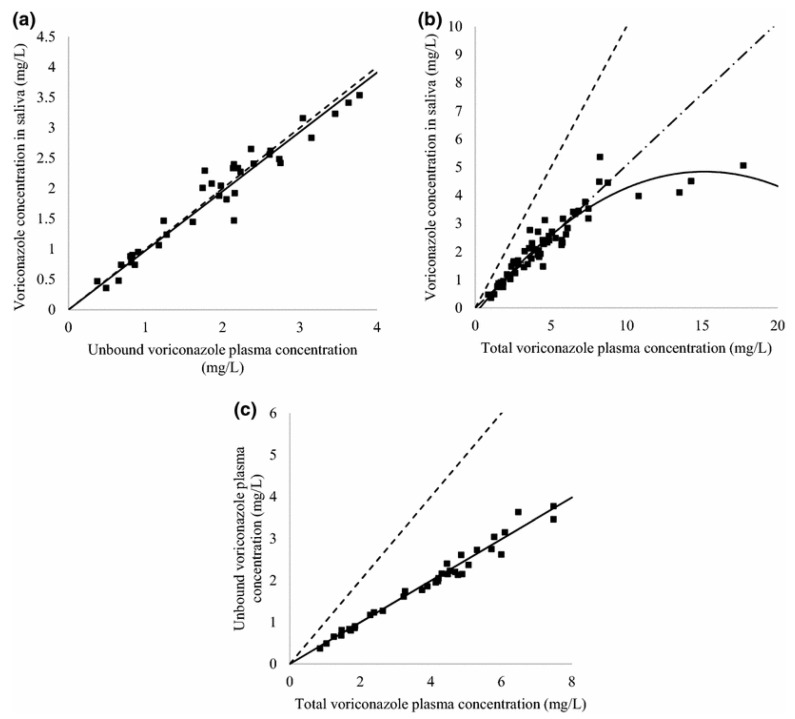
Scatterplots with linear mixed modeling to assess the agreement between voriconazole concentrations in saliva and unbound (**a**) and total (**b**) plasma voriconazole concentrations, and the agreement between unbound and total plasma voriconazole concentrations (**c**). The identity lines are presented as dashed lines, and the regression lines are depicted as solid lines. In panel (**b**), the dashed–dotted line represents the regression line when total plasma voriconazole concentrations above 10 mg/L are excluded [82].

In most cases, the determination of antifungal drugs was performed in plasma or serum samples. However, blood sampling can be sometimes difficult, especially in pediatric and ambulatory patients. For this reason, non-invasive samples for TDM are desirable. Vanstraelen et al. developed a method for the determination of voriconazole in saliva samples [82]. The authors compared results obtained for saliva samples with those obtained for plasma samples. Before analysis, samples (saliva or plasma) were diluted with Hanks’ Balanced Salt Solution supplemented with HEPES (4-(2-hydroxyethyl)-1-piperazineethanesulfonic acid) adjusted to pH 7.4. The samples after the addition of IS and NaOH were extracted using diethylether. Then, samples were centrifuged, and the organic layer was evaporated to dryness under a gentle stream of air. The residue was redissolved in a mixture of methanol and water. In the next step, samples were transferred into autosampler vials in order to be analyzed using LC-MS/MS. The LC separation was performed on a C18 column with a mobile phase containing methanol and water. Analytes were detected on triple quadrupole MS with ESI operated in positive mode. The LLOQ obtained by the procedure was 0.007 mg/L for both saliva and plasma samples. Concentrations of voriconazole in saliva were correlated with unbound and total plasma voriconazole concentrations (Figure 6). Therefore, saliva samples can be successfully used for TDM of voriconazole. 

Most separations of antifungal drugs were carried out on C18 columns. Sometimes for the analysis of these drugs, columns with other stationary phases such as pentafluorophenyl were applied. Triazole antifungal drugs-fluconazole, itraconazole, posaconazole, and voriconazole—were simultaneously analyzed in human plasma samples by LC-MS/MS [83]. For sample preparation, a mixture containing methanol and 0.2 M ZnSO_4_ was added to plasma samples for protein precipitation. The samples were after mixing and centrifugation injected into on-line combined SPE with LC-MS/MS. After injection, samples were loaded onto the Oasis HLB SPE column. A potentially interfering compound was washed using a mixture of methanol and water. Then, the analytes were eluted from the SPE column in back-flush mode and separated on the pentafluorophenyl (PFP) column with a mobile phase containing acetonitrile, water, and ammonium formate. Analytes were detected using triple quadrupole MS with ESI operated in positive ionization mode. After each analysis, the SPE column was washed with a mixture of acetonitrile and water. The LOD values were 10, 2, 3, and 10 ng/mL for fluconazole, voriconazole, posaconazole, and itraconazole, respectively. The LLOQs obtained by the proposed procedure were 14 ng/mL for all the investigated antifungal drugs. The usefulness for TDM of the proposed procedure was confirmed by the determination of investigated triazole antifungal drugs in 48 plasma samples obtained for patients treated with these drugs.

Other examples of the application of LC in the analysis of antifungal drugs are given in Table 4.

## 6. Antiviral Drugs

TDM is also very important in the optimization of antiviral treatments. The routine application of TDM for antiviral drugs could be useful especially in situations to assess compliance, to manage drug–drug interactions, to monitor antiretroviral drugs in pathophysiological status, e.g., virological failure, during pregnancy, and in application in children therapy. 

TDM is particularly desirable for monitoring of the concentration of antiviral drugs in HIV-infected patients. The main goal of the anti-HIV therapy is to prevent disease progression and to reduce morbidity and mortality. In treating patients infected by HIV, different classes of antiretroviral drugs have been developed to target various life cycles of the retrovirus. Drug classes include nucleoside reverse transcriptase inhibitors, non-nucleoside reverse transcriptase inhibitors, and protease inhibitors as well as two new classes: entry inhibitors and integrase strand transfer inhibitors [13]. The highly active antiretroviral therapy involves life-long treatment and requires constant dose optimization to fighting the development of viral resistance. Treatment is particularly difficult as each applied drug has large individual pharmacokinetic variability as well as important adverse effects, each drug can interact with other, and the complex dosing predisposes to non-compliance. TDM is also a useful tool in the clinical management of anti-hepatitis C virus drugs. The recommended treatment for hepatitis C virus-infected patients is based on PEGylated interferon and ribavirin. In recent years, telaprevir and boceprevir have been administrated in combination with PEGylated interferon and ribavirin. TDM of these drugs in hepatitis C virus-infected patients has significantly improved the management of the standard treatment. Accordingly, TDM of these antiviral drugs could be fundamental to optimize drug regimens by increasing efficacy and avoiding drug-related toxicity.

Most often, antiviral drugs concentrations are measured in plasma or dried plasma spots samples. Samples were often prepared by protein precipitation or LLE when LC-MS/MS was used for analysis and by protein precipitation followed by SPE when samples were analyzed by HPLC-UV. C18 columns using a mobile phase often contain acetonitrile, water, and various concentrations of formic acid. Another column, e.g., Phenyl-Hexyl, was used only in a few cases. MS/MS was commonly applied for the detection of various antiviral drugs. ESI has been the ion source of choice for most published methods. The majority of antiviral drugs can be detected by positive ionization mode. As combinations of different drugs are often used in antiviral therapy, analytical methods are usually developed for the simultaneous determination of several antiviral drugs.

### 6.1. HPLC-UV

HPLC-UV for the determination of antiviral drugs is rarely applied, because it often requires extensive sample preparation due to the limited specificity of the detection mode and the poor ultraviolet absorbance of some compounds. However, HPLC-UV procedures have been sometimes described. HPLC-UV represents a cheaper option and is easier to adapt to the clinical loboratories.

The HPLC-UV method was developed and validated for the simultaneous quantification of antiretroviral drugs: atazanavir, dolutegravir, darunavir, efavirenz, etravirine lopinavir, raltegravir, rilpivirine, and tipranavir in human plasma [92]. Before analysis, plasma samples underwent a heat inactivation procedure for HIV (35 min at 58 °C). After the addition of IS, samples were vortex-mixed, and then acetate buffer at pH 4.5 was added for protein precipitation. In the next step, samples were vortex-mixed and centrifuged. Subsequently, SPE was performed on C18 cartridges. Before sample loading, cartridges were activated with methanol followed by acetate buffer. Then, the cartridges were washed with acetate buffer, followed by water and then elution was carried out using methanol and acetonitrile solution. Eluted solution was evaporated, and the residue was resuspended in a mixture of acetonitrile and water, centrifuged, filtered, and then injected into the HPLC system. A C18 column and mobile phase containing acetonitrile and acetate buffer at pH 4.5 were applied for the separation of analytes. Investigated drugs detection was done by monitoring the absorbance at wavelengths of 260 nm for atazanavir, dolutegravir, darunavir, efavirenz, lopinavir, and tipranavir and at 305 nm for etravirine and raltegravir. LOQs ranged from 19.8 ng/mL for rilpivirine to 529 ng/mL for tipranavir. This method has been successfully applied to routine TDM and pharmacokinetics investigation in HIV-infected patients.

### 6.2. LC-MS

The practice of treating virus-infected patients with different types of combination drug therapies has led to the development of simultaneous LC-MS/MS methods. These methods have been developed to include a wide range of possible therapies, as each patient may be individualized to receive any combination of antiretroviral drugs.

The HPLC-MS/MS method was applied for the simultaneous quantification of antiretroviral drugs: dolutegravir, elvitegravir, rilpivirine, darunavir, ritonavir, and raltegravirand raltegravir-β-d-glucuronide in human plasma [93]. Before HPLC analysis, samples were prepared by protein precipitation with acetonitrile. After centrifugation, the samples were dried under a gentle stream of nitrogen. The dry residue was dissolved in the mobile phase before being transferred to an HPLC system. Analytes were separated on a phenyl-hexyl column with a mobile phase containing acetonitrile, water, and formic acid. Detection was performed on quadrupole MS operated in negative ESI mode for raltegravir-β-d-glucuronide or in positive ESI mode for the other investigated compounds. The method described has been successfully applied to determine the concentrations of antiviral drugs in plasma samples for the purpose of HIV-infected patients TDM.

**Figure 7:**
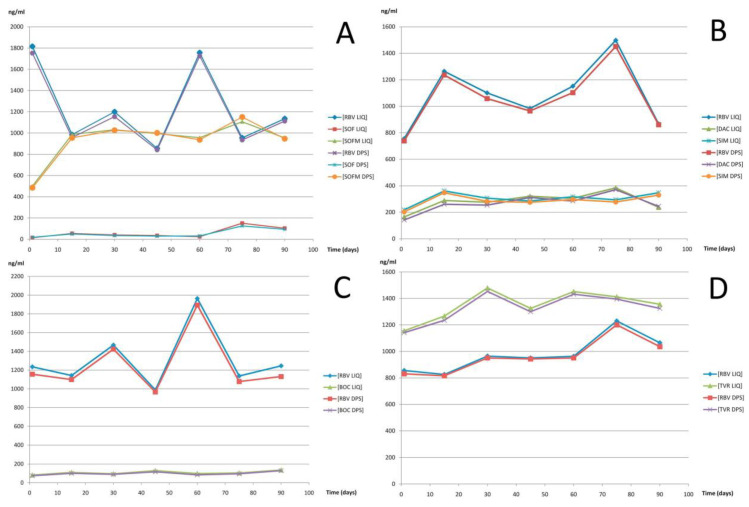
Time-course of the drug levels (measured both in liquid plasma and dried plasma spot (DPS) samples) in a patient under therapy. Time course of drug through concentrations in plasma samples from four different patients receiving different combined interferon-free antiviral therapies. Concentration of the drugs (ng/mL) are plotted vs. time (days) since the beginning of therapy. TDM was performed every other week for three months. (**A**). Blue diamond = liquid plasma ribavirin (RBV), purple cross = dried plasma spot (DPS) RBV, red square = liquid plasma sofosbuvir (SOF), light blue star = DPS SOF, green triangle = liquid plasma metabolite GS 331007 (SOFM) and orange circle = DPS SOFM concentration, respectively. (**B**). Blue diamond = liquid plasma RBV, red square = DPS RBV, green triangle = liquid plasma Daclatasvir (DAC), purple cross = DPS DAC, light blue star = liquid plasma Simeprevir (SIM) and orange circle = DPS SIM concentration respectively. (**C**). Blue diamond = liquid plasma RBV, red square = DPS RBV, green triangle = liquid plasma Boceprevir (BOC), purple cross = DPS BOC concentration, respectively. (**D**). Blue diamond = liquid plasma RBV, red square = DPS RBV, green triangle = liquid plasma Telaprevir (TVR), purple cross = DPS TVR concentration, respectively [94].

Conti et al. reported an LC-MS/MS method for the determination of anti-hepatitis C virus drugs: the classical nucleoside analog ribavirin; the first-generation viral non-structural protease NS3/4A inhibitors telaprevir and boceprevir; the more recent NS3/4A inhibitor simeprevir; the NS5A inhibitor daclatasvir; the NS5B inhibitor sofosbuvir; and its GS331007 metabolite in plasma and in dried plasma spots [94]. For sample preparation, plasma was mixed with IS working solution, and then, deproteinizing solution (1.78 g of ZnSO_4_·7 H_2_O dissolved in 100 ml of H_2_O–methanol 20–80 *v/v*) was added. Next, samples were vortexed and centrifuged; then, the clear supernatant was diluted with a mobile phase. Subsequently, samples were vortexed and mixed before injection into the HPLC system. The whole dried plasma spots were punched out, cut to smaller pieces with clean scissors, and all the pieces were transferred into an Eppendorf vial. The same deproteinizing solution used for liquid plasma was added. The samples were vortexed and centrifuged. The clear supernatant was diluted with a mobile phase and introduced into the HPLC system. Investigated anti-hepatitis C virus drugs possess various chemical properties; therefore, analysis was performed on a Chromsystems MasterColumn. The column is dedicated for the simultaneous analysis of compounds with various chemical properties. For the elution of analytes, a mobile phase composed of methanol, water, acetic acid, and ammonium acetate was applied. QTRAP MS with an ESI source operated in positive ionization mode was coupled to HPLC for the detection of investigated drugs. Obtained LODs values were 6 (± 3.2), 6 (± 5.9), 5 (± 4.4), 7 (± 5.4), 7 (± 4.9), 6 (± 4.8), and 6 (± 6.4) ng/mL for ribavirin, the metabolite of sofosbuvir, sofosbuvir, daclatasvir, boceprevir, telaprevir, and simeprevir, respectively. LOQs were 10 (± 1.2), 10 (± 4.9), 10 (± 4.4), 10 (± 4.4), 10 (± 6.4), 10 (± 3.4), and 10 (± 6.4) ng/mL for ribavirin, the metabolite of sofosbuvir, sofosbuvir, daclatasvir, boceprevir, telaprevir, and simeprevir, respectively. Figure 7 shows a graphical presentation of TDM obtained for patients receiving different combined antiviral therapies monitored along a 3-month period with sampling every other week. The proposed method enables the simultaneously determination of drugs with different chemical properties.

Additionally, examples of sample preparation and chromatographic procedures for antiviral drugs are listed in Table 5.

## 7. Cardiovascular Drugs

Cardiovascular diseases are a major problem in the human population. The incidence of cardiovascular diseases has increased in recent decades and it has been estimated that these diseases are the most common cause of death in many countries. For this increasing problem, an intensive development of cardiological pharmacotherapy has been observed in recent years, and new drugs with different mechanisms of action have been investigated to improve the effectiveness of combination therapy. The most common cardiac diseases are hypertension, angina pectoris, cardiac arrhythmias, glaucoma, migraine headaches, and thyrotoxicosis. The inter-individual differences in the action and rate of drugs’ biotransformation are the result of the inter-relationship between environmental factors, e.g., illness, other medications, exposure to other xenobiotics, diet, genetic factors such as genotype, gender, and race, and individually diverse bioavailability. The determination of drug concentrations during therapy is necessary to determine correlations between drug concentrations in the blood and their effects, especially when the pharmacokinetics of the drugs are complex and vary between individuals. For these reasons, it is necessary to develop new analytical procedures for analyzing these compounds in biological samples.

The determination of cardiovascular drugs was mostly performed in human serum samples. Samples containing these drugs were most often prepared by LLE using various solvents or a mixture of solvents often containing ethers or ethyl acetate. C18 columns were commonly utilized for the separation of cardiovascular drugs. Mobile phases most often contained an organic modifier, water, and additions of salts such as ammonium acetate or formate or acids, commonly formic acid. Analytes most often detected using MS detection mostly involved ESI operated in the positive ion mode.

### 7.1. HPLC-UV

Sometimes, HPLC-UV was applied for the determination of cardiovascular drugs in samples from cardiac patients. 

For analysis of very polar drugs, columns with alkylbonded stationary phases (C18 or C8) and mobile phases containing ion-pairing reagents were successfully applied. Propafenone and its metabolites 5-hydroxypropafeone and *N*-depropylpropafenone were simultaneously determined in human serum by LC-UV [100]. Before chromatographic analysis, IS was added to the serum samples. Then, the samples were alkalinized using 0.02 M sodium carbonate and were treated with diethyl ether three times. After vortex-mixing, samples were centrifuged, and then, an organic layer was evaporated to dryness under a stream of nitrogen. The residue was reconstituted with the mobile phase and transfered into the LC system. Analytes were separated on a C18 column with a mobile phase containing acetonitrile, water, acetic acid, and 1-pentanesulfonic acid sodium salt used as an ion-pairing reagent. The UV detection wavelength was set at 250 nm. The LOQs were 3.0, 1.4, and 1.4 ng/mL for propafenone, 5-hydroxypropafeone, and *N*-depropylpropafenone, respectively.

### 7.2. HPLC-FLD

Several HPLC-FLD procedures with and without a derivatization process have been reported for the quantification of cardiovascular drugs in biological samples.

Rosuvastatin was determined in human serum by HPLC-FLD with pre-column derivatization [101]. Serum samples were prepared by the addition of IS solution and mixing. Then, the acetate buffer and ethyl acetate and methyl tertiary buthyl ether were added, and samples were vortexed. Next, the organic layer was separated and dried using anhydrous Na_2_SO_4_. After the separation of anhydrous supernatant, samples were evaporated to dryness under nitrogen. The residues were further subjected to the derivatization procedure. The derivatization was performed with 9-anthryldiazomethane solution. The samples were adjusted with acetonitrile, and the solution was kept in the dark for 1 h at ambient temperature. Finally, the reaction mixture was injected into the HPLC system. Chromatographic analysis was carried out on a C18 column with a mobile phase containing acetonitrile and water. Rosuvastatin was revealed with a fluorescence detector; excitation and emission wavelengths were set respectively at 366 and 410 nm. The LOD value was 0.68 pg/mL, and the LOQ value was 2.3 pg/mL. The application of the pre-column derivatization process and fluorescence detection allowed for the determination of rosuvastatin at very low concentration levels. The suitability of the developed methods for clinic application was confirmed by determination of the drug in plasma samples obtained from healthy volunteers after the oral administration of 20 mg of rosuvastatin.

Rarely, the determination of enantiomers of chiral drugs in biological samples was described. Flecainide, a chiral compound administered as a racemic mixture for the treatment of supraventricular tachyarrhythmias in human serum and urine was analyzed by HPLC-FLD [102]. The detection of flecainide by FLD does not require a pre-column derivatization process. For sample preparation, serum and urine after the addition of IS were alkalinized using 0.02 M sodium carbonate and subsequently treated with diethyl ether. Then, the samples were vortex-mixed and centrifuged. The organic layer was evaporated to dryness under a stream of nitrogen. The residues were reconstituted with mobile phase solution and injected into the chromatographic system. A polysaccharide-based chiral column and mobile phase containing acetonitrile, water, and 0.1 M potassium hexafluorophosphate was applied for chromatographic analysis. Detection was performed by fluorescence detector operating at an excitation wavelength of 300 nm and emission wavelength of 370 nm.

### 7.3. LC-MS/MS

LC-MS/MS is an increasingly important tool in cardiovascular drugs TDM as it offers increased sensitivity and specificity compared to other methods, and in some cases, it may be the only viable method for quantifying drugs without natural chromophores or fluorophores.

One of the drugs that requires special monitoring of its concentration in patients is digoxin. Digoxin has a positive inotropic and batmotropic impact on heart muscle, but it has also negative chronotropic and dromotropic effects [103]. There is a small variation between the therapeutic and toxic dosage of the drug. The prescription dose of digoxin is 1−2 ng/mL, and more than 2.8 ng/mL of this medication cause toxicity. The LC/MS/MS method has been described for the determination of digoxin in human serum [104]. Samples were prepared by LLE with methyl tert-butyl ether. A C18 column and mobile phase containing acetonitrile, water, ammonium acetate, and formic acid were applied for chromatographic separation. Detection was carried on QTRAP MS operated in positive ionization mode. The LLOQ of 0.1 ng/mL was achieved for digoxin. The described procedure was applied to determine the serum digoxin concentrations from heart failure patients receiving a dose of 0.125 mg/day of digoxin. The digoxin concentrations in 37 samples were found to be higher than the target range, while those for 4 samples were lower. Therefore, therapeutic drug monitoring of digoxin is very important for dosage adjustment regimens in order to obtain desirable therapy outcomes.

**Figure 8:**
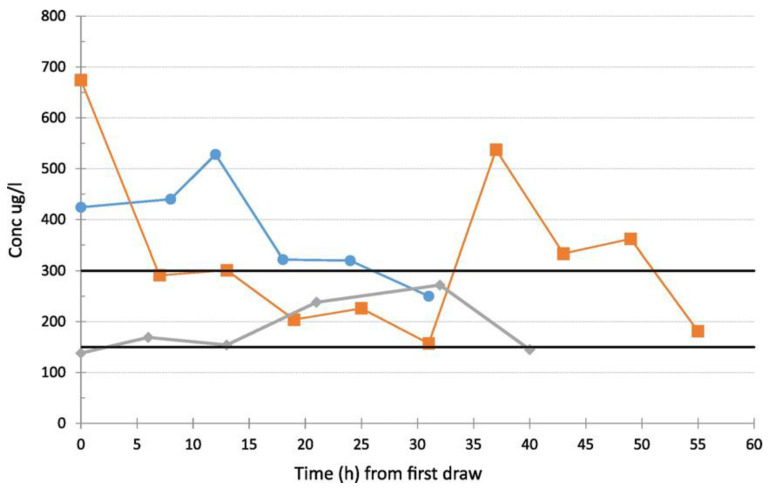
Milrinone serum concentration time profiles for 3 patients post-cardiac surgery (marked in orange, blue, grey). Solid black line at 150 and 300 μg/L indicate therapeutic range [105].

Milrinone is a bipyridine phosphodiesterase inhibitor with positive inotropic and vasodilatory effects, whose concentration in patients should be monitored, especially in case of longer-term treatment. It is essential owing to a narrow therapeutic range, an increased half-life in renal failure, and toxicity associated with high levels. Since milrinone is used in intensive care medicine in patients with compromised cardiac function, and the drug has a short half-life in organisms, is reliable, and is able to provide results within several hours, analytical methods should be developed. Milrinone is also used in therapy of low cardiac output syndrome post-cardiac surgery and a second-line therapy in children with vasoconstricted septic shock. Raizman et al. quantified milrinone in serum from pediatric patients by LC-MS/MS [105]. Serum samples were prepared by liquid–liquid extraction with ethyl acetate. Following centrifugation, the supernatant was evaporated under a stream of nitrogen gas at room temperature until completely dry. Samples were then reconstituted in mobile phase, vortexed, centrifuged, and injected to the HPLC system. Separation was performed on a C18 column with a mobile phase containing methanol, water, and ammonium formate. MS detection was conducted using triple quadrupole with ESI operated in positive ion mode. The LLOQ obtained by the procedure was 22 μg/L. Figure 8 presents milrinone serum concentration time profiles determined by the proposed analytical procedure for 3 patients post-cardiac surgery. Two patients had milrinone levels well above the therapeutic range at the beginning of treatment, but they were then titrated down to the target therapeutic range. In the third patient, milrinone levels were at the lower end of the therapeutic range, their dose was titrated up, and levels were maintained within the target therapeutic level.

Coronary heart disease is the result of coronary atherosclerosis, whose pathogenesis is the deposition and retention of cholesterol in arterial walls. Statins have assumed the central role in the treatment of hypercholesterolaemia. Statins specifically inhibit the enzyme HMG-CoA reductase, thereby preventing the conversion of HMG-CoA to mevalonate, which is an early rate-limiting step in cholesterol biosynthesis. Besides lipid lowering activity, statins improve endothelial function, enhance the stability of atherosclerotic plaques, decrease oxidative stress, and inhibit the thrombogenic responses [6]. Yin et al. reported the LC-MS/MS method for the quantification of pitavastatin in human plasma [106]. Plasma samples before analysis were prepared by the LLE technique. Samples after the introduction of IS and phosphoric acid were vortexed and then a mixture of ether and dichloromethane were added. After vortexing, samples were centrifuged and the organic layer was evaporated to dryness under a gentle stream of nitrogen. The residue was dissolved in mobile phase and injected into an LC-MS/MS system. A C18 column and mobile phase containing acetonitrile, methanol, water, and formic acid were used for analysis. Detection was carried out on triple-quadrupole MS with an ESI source operated in positive mode. The LLOQ obtained by the described procedure was 0.2 ng/mL. Additional examples of sample preparation and chromatographic procedures for cardiovascular drugs are listed in Table 6.

## 8. Psychotropic Drugs

Psychotropic drugs are used to treat psychosis such as schizophrenia, bipolar disorder, and clinically defined depression and other mental disorders. Psychotropic drugs are divided into different groups according to their major clinical indication: antidepressant drugs, antipsychotic drugs, mood-stabilising drugs, anxiolytic drugs, antidementia drugs, drugs for the treatment of substance-related disorders, and drugs for the treatment of attention deficit disorder [110]. Major classes of antidepressants include tricyclics, monoamine oxidase inhibitors, and selective serotonin re-uptake inhibitors. Selecting the adequate psychotropic drugs and the right dosage for patients with psychological illnesses is a long process that may be caused by pharmacodynamic processes and could also be due to the individual variability in the pharmacokinetics of the drugs. Plasma or serum concentrations of antipsychotics in various patients vary strongly with the same doses. Selecting the adequate drug and the right dosage for patients with psychological illnesses is a long process taking often several weeks or months. Many psychotropic drugs such as tricyclic antidepressants have a narrow therapeutic window with a large intra- and inter-individual variation in metabolism and clearance. Additionally, patients are often treated with a combination of psychiatric drugs, thus increasing the risk of drug–drug interactions. Psychiatry was one of the first medical disciplines introducing routine TDM practice. TDM in psychiatry is advisable for patients with polypharmaceutical treatment, patients with pharmacokinetically relevant comorbidities, patients with genetically determined pharmacokinetic abnormalities and geriatric patients. In most cases, it is recommended to monitor both parent and metabolite compounds, as both can contribute to the therapeutic and toxic effects. Thus, TDM enables achieving a rapid response to treatment and therefore increases the safety of antipsychotic treatment [111]. Often, metabolites of psychotropic drugs are active compounds and are simultaneously determined with parent drugs in biological samples. The simultaneous determination of psychotropic drugs and their metabolites in biological fluids presents many difficult analytical problems due to their low analyte concentrations, demanding greater assay selectivity and sensitivity, the complex matrix of biological samples, and frequently, their strong binding to parent compounds [11]. Therefore, reliable analytical methods for the quantification of psychotropic drugs are important for clinical laboratories. Currently, the leading analytical method for the determination of psychotropic drugs is liquid chromatography coupled with sensitive detection methods, especially tandem mass spectrometry.

Psychotropic drugs and their metabolites were commonly quantified in serum or plasma samples. Increasingly, alternative, less invasive matrices such as saliva or dried blood spots are used. In the literature, LLE and less often SPE procedures are the most reported methods for sample preparation before chromatographic analysis of psychtropic drugs and their metabolites in biological matrices. Most often, psychotropic drugs were analyzed on columns with alkyl stationary phases, most often C18 and rarely C8. Several procedures featuring the application of phenyl stationary phases for the separation of these compounds have also been described. In most reported cases, mobile phases at acidic condition (addition of acids or buffers at acidic pH) were used for the analysis of psychotropic drugs and their metabolites. The application of mobile phases with the addition of amines as silanol blockers was also sometimes reported. For the detection of psychotropic drugs, various detection techniques were used, most often UV and especially MS. 

### 8.1. HPLC-UV

Owing to the chemical structure of most psychotropic drugs and their metabolites having UV light absorption properties, relatively many HPLC-UV procedures were developed for the analysis of the compounds of interests in various biological samples.

**Figure 9:**
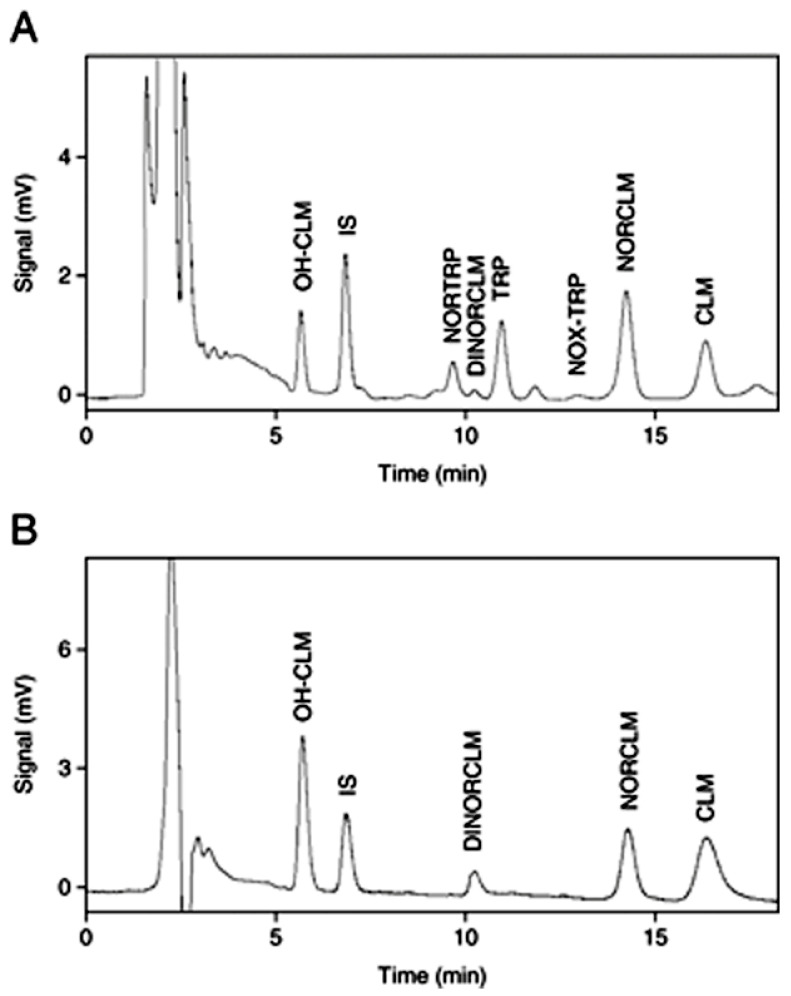
Chromatograms of plasma samples from patients subjected to therapy with tricyclic antidepressants. Stationary phase: C8 column (150 × 4.6 mm particle size (id), 5 μm); mobile phase: ACN/pH 3.0, 50 mM phosphate buffer (32:68 *v*/*v*); flow rate: 1 mL/min; detection: 220 nm; loop: 50 μL [113]. Abbreviations: 8-hydroxyamoxapine (OH-CLM), internal standard (IS), nortriptyline (NORTRP), *N,N*-didesmethylclomipramine (DINORCLM), amitriptyline (TRP), triprolidine N-oxide (NOX-TRP), *N*-desmethylclomipramine (NORCLM), clomipramine (CLM) (**A**). Chromatogram obtained for plasma samples from a patient undergoing polypharmacy with triprolidine and clomipramine; (**B**). chromatogram obtained for plasma samples from a patient undergoing monotherapy with clomipramine [112].

Mercolini et al. reported an HPLC-UV method for the simultaneous determination of seven tricyclic antidepressants and seven metabolites in human plasma [112]. Before chromatographic analysis, plasma samples were prepared by an SPE procedure performed on C2 cartridges. Cartridges were activated with methanol and then conditioned with ultrapure water. To plasma samples, IS working solution and ultrapure water were added, and the resulting mixture was loaded onto a previously conditioned cartridge. Next, the cartridges were washed twice with ultrapure water, once with a mixture of methanol and water and once with methanol. Then, the analytes were eluted with methanol. The eluate was dried under vacuum, re-dissolved with mobile phase, and then injected into the chromatographic system. A C8 column and mobile phase containing acetonitrile and phosphate buffer at pH 3.0 were applied for the separation of analytes. The UV detection of investigated drugs and metabolites was performed at λ = 220 nm. The LOD values were in the 0.2–3.0 ng/mL range while the LOQ values were between 0.5 and 9.0 ng/mL for all analytes. The proposed analytical procedure is suitable for the TDM of patients treated with tricyclic antidepressants under monotherapy or polypharmacy regimens. Some examples of the results obtained by the described procedure are reported in Figure 9A,B, corresponding to plasma samples from a patient undergoing polypharmacy with TDM and clomipramine (Figure 9A) and a patient undergoing monotherapy with clomipramine (Figure 9B).

Increasingly, psychotropic drugs are determined in saliva samples whose collection is non-invasive and painless. Venlafaxine, an antidepressant of the selective serotonin–norepinephrine reuptake inhibitor class and its main metabolite, *O*-desmethylvenlafaxine were quantified in human saliva by HPLC-UV [113]. Before chromatographic analysis, saliva samples were centrifuged and then extracted with dichloromethane. After centrifugation, the dichloromethane layer was transferred to a glass tube and evaporated. The residue was re-dissolved in a mixture of acetonitrile and water and then injected into the HPLC-UV system. Separation was performed on a C18 column using a mobile phase containing acetonitrile and phosphate buffer. The UV detection wavelength was set at 226 nm. The values of LOD were 3.1 and 2.8 ng/mL for venlafaxine and *O*-desmethylvenlafaxine, respectively. The LLOQ were set at 10.2 and 9.4 ng/mL for venlafaxine and *O*-desmethylvenlafaxine, respectively. Applicability of the procedure was confirmed by analysis of the saliva samples obtained from depressed patients treated with venlafaxine.

Protonated basic psychotropic drugs can easily interact with residual silanol groups of the alkylbonded stationary phases. Thus, besides the reversed phase retention mechanism, an ion-exchange retention mechanism occurs as well, which often results in an asymmetry of peaks, irreproducible retention, poor efficiency, and worse separation. The silanol ion-exchange interaction can be reduced by using a mobile phase at low pH, when the silanol ionization is suppressed, a mobile phase at high pH to suppress solutes ionization, and the addition of anionic ion-pairing reagents, making neutral associates or the addition of organic amines as silanol blockers. Good results were also obtained by selecting a stationary phase to minimize the interaction between analyte and residual silanols such as cyanopropyl, phenyl, and pentafluorophenyl stationary phases with hydrophobic π–π active aromatic moieties, which generate a concerted π–π reversed-phase retention mechanism that diversivies the common reversed phase( RP)-interaction properties. 

The simultaneous quantification of six beta-blockers (metoprolol, timolol, bisoprolol, propranolol, carvedilol, and nebivolol), three of their metabolites (α-hydroxy metoprolol, *N*-desisopropyl propranolol, and 4′-hydroxy carvedilol), three antipsychotics (olanzapine, clozapine, and quetiapine) and three of their metabolites (*N*-desmethyl olanzapine, *N*-desmethyl clozapine, and *N*-desalkyl quetiapine) in human serum were performed by HPLC-UV [114]. Serum samples were prepared by centrifugation. Analytes were separated on a Phenyl-Hexyl column using a mobile phase containing acetonitrile, methanol, and dihydrogen phosphate buffer (pH 3.1). Investigated compounds were detected at various wavelengths: 215, 226, 242, and 299 nm. Obtained LOD values were from 0.91 ng/mL for 4′-hydroxy carvedilol to 15.79 ng/mL for nebivolol. LOQs were from 1.25 ng/mL for *N*-desisopropyl propranolol and propranolol to 40 ng/mL for nebivolol. This analytical procedure was successfully applied for TDM and the analysis of these drugs in enzyme kinetic experiments to clarify the pharmacokinetic interaction between antipsychotics and often simultaneously administrated β-blockers.

### 8.2. HPLC-FLD

Several HPLC-FLD procedures for the determination of psychotropic drugs in biological samples have also been reported, especially often for the determination of fluoxetine.

**Figure 10:**
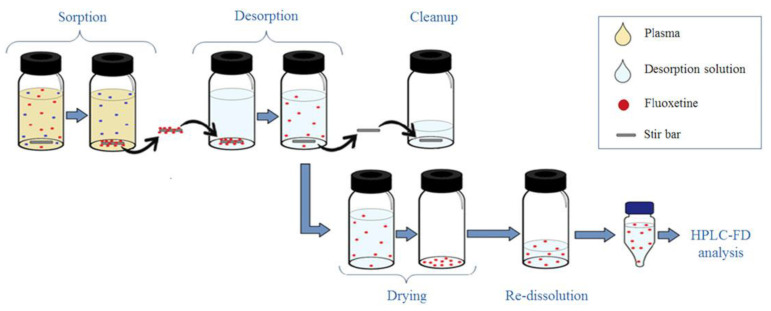
Extraction procedure of fluoxetine by stir bar sorptive extraction (SBSE) in plasma [115].

Fluoxetine was analyzed in human plasma samples by stir bar sorptive extraction coupled with high-performance liquid chromatography–fluorescence detection (SBSE–HPLC–FLD) [115]. The volumes of plasma samples, buffer solution, and extraction solvents were optimized and compared with those used in previous investigations described in the literature. In the proposed procedure, SBSE bar and sodium borate buffer (pH 9.00) were added to plasma. The dilution of samples in borate buffer at pH 9.0 was used to maximize the extraction of the fluoxetine from the plasma, because in this condition, the analyte is partially nonionized (pKa 10.5), and a good partition in the polydimethylsiloxane polymeric phase is achieved. The authors also compared different desorption modes and obtained fluoxetine concentrations that were significantly higher when magnetic stirring was applied in comparison with previously used ultrasonic desorption. On the basis of the results, the sorption step was performed employing a multipoint magnetic stirrer. The mixture of methanol and acetonitrile was applied as desorption solution. A schematic illustration of the extraction procedure is presented in Figure 10. Analysis was carried out on a C8 column with a mobile phase containing acetonitrile and sodium phosphate buffer (pH = 3.07). The FLD operating at an excitation wavelength of 230 nm and emission wavelength of 290 nm was used for the detection of fluoxetine. The values of LOD and LOQ were 9.80 and 32.67 ng/mL, respectively. The procedure was successfully applied for the determination of fluoxetine in plasma samples from patients.

### 8.3. HPLC-ED

Electrochemical detection is an alternative detection mode for the monitoring of psychotropic drugs and their metabolites that was frequently coupled with HPLC. 

Riman et al. developed micro carbon pencil lead electrode for high-performance liquid chromatography coupled with electrochemical detection (HPLC-ED) analysis of selected antipsychotic drugs (quetiapine, olanzapine, clozapine, fluphenazine, promethazine, levomepromazine, chlorpromazine, thioridazine, and perphenazine) [116]. For sample preparation, protein precipitation acetonitrile was applied. Chromatography was carried out on a C8 column using a mobile phase containing acetonitrile, methanol, water, and K_2_HPO_4_ at pH 7.5. For detection, an electrochemical detector employing the utilizing non-standard, 0.2 mm diameter pencil lead as an in-capillary working electrode was connected to HPLC. The detector allows for the determination of nine selected antipsychotic drugs at physiological concentration levels. All investigated drugs exhibit electrochemical activity manifested by well-defined anodic waves in cyclic voltammetry at the pencil graphite electrode. LODs obtained by the described method were in a range from 1.0 nmol L^−1^ for olanzapine to 7.9 nmol L^−1^ for chlorpromazine; LOQs were in a range from 3.4 nmol L^−1^ for olanzapine to 26.2 nmol L^−1^ for chlorpromazine. The advantage of the application of an innovative electrochemical detection detector is the lowered flow rate; therefore, the use of a mobile phase with reduced organic modifier content offers greener analysis.

### 8.4. LC-MS/MS

Most analyses of psychotropic drugs in various biological samples were performed by LC-MS/MS procedures. 

A fast, simple LC-MS/MS method for the quantitative detection of antidepressant drugs and their metabolites in plasma for use in therapeutic drug monitoring and routine forensic analysis without the need for any time-consuming sample preparation was developed for the simultaneous quantification of 40 compounds in human plasma [117]. Samples were prepared by the addition of 1M carbonate buffer (pH 9.5) and methyl-tertiarybutyl-ether followed by vortex-mixing. Afterwards, the upper layer was separated from the aqueous one by centrifugation and evaporated to dryness under nitrogen. The residue was reconstituted in acetonitrile, then vortex mixed and transferred into an HPLC system. The separation of analytes was carried out on a C8 column using a mixture of acetonitrile, water, and formic acid as a mobile phase. Investigated compounds were detected by triple quadrupole MS with ESI operated in positive ionization mode. The described procedure allowed the simultaneous quantification of 40 antidepressants or their metabolites in plasma samples in a relatively short chromatographic run after simple sample clean-up based on LLE. 

The application of TDM practice for the treatment by psychotropic drugs is especially important in pregnant women. Sertraline was quantified in maternal blood, amniotic fluid, and umbilical cord blood by LC-MS/MS [118]. Serum was prepared by the centrifugation of blood samples. Separation was performed on a UPLC C18 column. For detection, triple quadrupole MS was used. Daily doses were correlated with maternal serum and umbilical cord blood concentrations, and serum levels were correlated with levels in amniotic fluid. The investigations indicated that maternally administered sertraline is permanently accessible to the fetus via amniotic fluid. The investigations on sertraline concentrations in amniotic fluid gave evidence that sertraline administered for pregnant women is constantly accessible to the fetus via amniotic fluid. However, a relatively low penetration into fetal circulation may contribute to a sufficient safety profile of sertraline during pregnancy.

**Figure 11:**
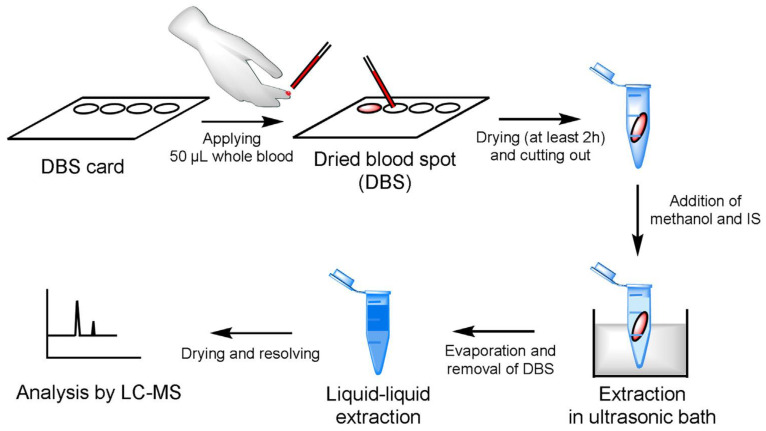
Dried blood spot extraction and sample purification workflow [111].

The minimally invasive dried blood spots sampling technique is recognized as a suitable alternative for conventional sampling methods, as TDM interventions should be applied in the most cost-effective, rational, and clinically useful manner. A method for the simultaneous quantification of four antipsychotics and antidepressants: citalopram, mirtazapine, risperidone, and its active metabolite 9-hydroxyrisperidone in dried blood spot using HPLC-MS for therapeutic drug monitoring was described [111]. For sample preparation, the analytes from dried blood spots were extracted by methanol using an ultrasonic bath. After drying under a nitrogen stream, the obtained residue was purified twice by liquid–liquid extraction with a mixture of hexane with butyl acetate and sodium hydroxide solution in water. The samples were shaken, centrifuged, and frozen. After freezing of the aqueous layer, the organic phase was decanted. Subsequently, the organic layers of both extraction steps were merged and evaporated to dryness under nitrogen. The residue was reconstituted in methanol and transferred into an LC-MS system. A scheme of the sample preparation workflow is presented in Figure 11. The separation of analytes was performed on a C18 column with a mobile phase containing acetonitrile and ammonium formate (pH = 3.5). Investigated psychotropic drugs were detected by MS with an ESI source operated in positive ionization mode. The correlation between serum and capillary blood concentration in patients to obtain a capillary blood/serum concentration ratio was also investigated. The authors showed that the method is suitable for the analysis of citalopram and mirtazapine in dried blood spot from capillary blood samples from polymedicated patients for TDM.

Thirty-one psychoactive drugs, including antidepressants, were determined in serum samples by UPLC coupled with high-resolution mass spectrometry (HRMS) [119]. Samples were prepared by the addition of acetonitrile for protein precipitation. After vortex-mixing and centrifugation, samples were cleaned by using Turbo-Flow technology and then injected into a chromatographic system. The separation of analytes was performed on a phenyl column with a mobile phase containing methanol, water, formic acid, and ammonium formate. For the detection of investigated psychotropic drugs, Orbitrap ESI-MS operated in positive ionization mode was applied. The authors compared the results obtained by LC coupled with high-resolution mass spectrometry with those obtained by a validated triple quadrupole-based quantification method used in a routine clinical laboratory. They indicated that high-resolution mass spectrometers are appropriate for the accurate and reliable quantification of analytes for TDM measurements. Other samples of chromatographic application methods for psychotropic drugs analysis are presented in Table 7.

## 9. Antiepileptic Drugs

Epilepsy is the most common serious neurologic disorder, where seizures result from electrical discharges in the brain. The pharmacotherapy with antiepileptic drugs is currently the therapeutic approach of choice regarding epilepsy, as it controls the occurrence of unpredictable epileptic seizures on approximately two-thirds of the patients [128]. Monotherapy is the standard initial therapy of epilepsy; adjunctive therapy including multiple antiepileptic drugs is one of the primary choices after the first antiepileptic drugs failed due to insufficient efficacy [129]. The most often prescribed drugs for first epilepsy treatment are carbamazepine, oxcarbazepine, lamotrigine, and phenytoin, which are the hepatic enzyme inducers. These drugs induce multiple p450 enzymes, increasing the clearance of drugs metabolized by these enzymes. Valproate inhibits the metabolism of carbamazepine epoxide (active carbamazepine metabolite) and lamotrigine, causing an increase of the serum concentrations of these compounds. Oxcarbazepine and topiramate are selective hepatic inhibitors. They inhibit CYP2C19, which is the main metabolite enzyme of phenytoin and may cause an increase in phenytoin concentration in an adjunctive therapy. Antiepileptic therapy is usually prophylactic and lifelong, and the relationship between dose and drug concentrations is unpredictable. The application of monitoring of drugs concentrations in patients allows for the personalization and optimization of the therapy, minimizing side and toxic effects, increasing the efficacy of the treatment, and reducing its costs due to fewer hospitalizations and a more rational use of drugs. For these reasons, the application of TDM for antiepileptic drugs is advisable, especially for patients receiving combined therapy. However, TDM in epilepsy is very complex and difficult. Due to the episodic nature of the condition, assessment of the clinical efficacy of antiepileptic drugs is especially challenging, as it is difficult to assess if the patient is responding to the therapy or is just free of seizures [10]. Additionally, many times, there are difficulties to differentiate clinical symptoms and signs of toxicity. To effectively optimize the treatment outcome and reduce the incidence of side effects, drug doses and scheduling should be personalized. A reliable TDM regimen should be established, especially when the patient is subjected to polypharmacy.

Therefore, the availability of rapid, sensitive, simple, accurate, and reliable analytical methods is essential to support TDM in clinical routine. Immunoassays and chromatographic techniques such as HPLC-UV, HPLC-DAD, and especially LC-MS/MS were most often applied for the monitoring of antiepileptic drugs concentrations in biological fluids for TDM.

The majority of described procedures for the analysis of antiepileptic drugs utilize plasma or serum as the bioanalytical matrix. Dried blood spots are recognized as a suitable alternative for conventional plasma or serum samples. Sometimes, these drugs were also determined in non-invasive saliva samples. Various procedures were used for the preparation of samples containing antiepileptic drugs. Sometimes, samples were prepared only by protein precipitation, most often with acetonitrile or methanol. More often, after protein precipitation, samples were prepared by SPE or LLE procedures. Several MEPS procedures for the preparation of samples containing antiepileptic drugs have also been reported. Analytes were commonly separated on C18 columns using mobile phases containing organic modifier, water, and additions of acids, acidic buffers, and sometimes salts. Mobile phases containing an addition of amines, e.g., triethylamine were also often applied for the analysis of antiepileptic drugs. The detection of antiepileptic drugs was relatively often performed by UV and rarely by FLD detection. MS detection was also increasingly applied. 

### 9.1. HPLC-UV

Several HPLC methods coupled to ultraviolet UV detection or DAD have been developed and validated for the determination of antiepileptic drugs and some of their main metabolites in biological samples from patients.

HPLC-UV was developed for the simultaneous determination of antiepileptic drugs—phenobarbital, primidone, phenytoin, carbamazepine, lamotrigine, oxcarbazepine—and some of their main metabolites, carbamazepine-10,11-epoxide, 10,11-trans-dihydroxy-10,11-dihydrocarbamazepine, and licarbazepine in human plasma [130]. Before HPLC-UV analysis to human plasma samples, IS working solution was added; then, samples were mixed with methanol in order to precipitate plasma proteins. After centrifuging, the supernatant was evaporated under a gentle nitrogen stream. Then, the residual volume of the supernatant was diluted with water and vortex-mixed. In the next step of sample preparation, the SPE was performed on the Oasis® HLB cartridge, which was previously conditioned with methanol, acetonitrile, and then water–acetonitrile. The loaded cartridge was washed four times with water. After drying the sorbent under airflow, analytes were eluted with ethyl acetate. Then, the samples were evaporated to dryness under a gentle stream of nitrogen gas, reconstituted with mobile phase by vortexing and ultrasonication, and injected into the HPLC system. A C18 column and mobile phase containing acetonitrile, methanol, water, and triethylamine were employed for chromatographic separation. Analytes were detected at λ = 237 nm. The LOQ values obtained by the analytical procedure were 0.50 µg/mL for phenytoin, 0.40 µg/mL for primidone, 0.25 µg/mL for phenobarbital and 10,11-trans-dihydroxy-10,11-dihydrocarbamazepine, 0.15 µg/mL for licarbazepine and 0.10 µg/mL for oxcarbazepine, carbamazepine, carbamazepine-10,11-epoxide, and lamotrigine. The proposed procedure was applied for the analysis of plasma samples from patients treated with phenobarbita phenytoin and carbamazepine.

**Figure 12:**
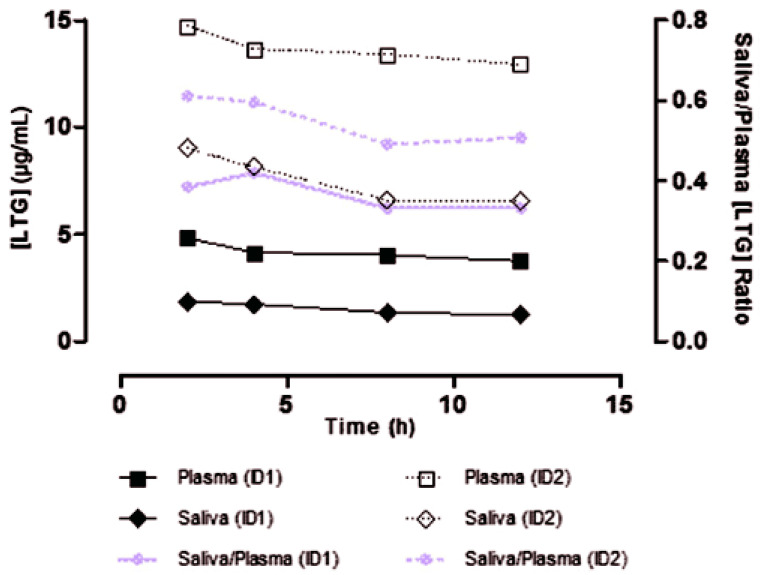
Concentration–time profiles of lamotrigine (LTG) obtained from plasma and saliva samples collected at 2, 4, 8, and 12 h post-dose (taking as reference the morning dose) in two patients (ID1 and ID2) under oral LTG therapy (ID1, 100 mg once-daily in the morning; ID2, 150 mg in the morning, and 200 mg at night in cotherapy with valproic acid). The corresponding salivary to plasma LTG concentration ratios were also calculated at 2, 4, 8, and 12 h post-dose and graphically represented for both patients [131].

The HPLC-DAD method was applied for the determination of lamotrigine, a broad-spectrum antiepileptic drug used as monotherapy or in add-on therapy regimens in adults and children [131]. The authors described procedures for determination of the drug concentrations in the human plasma and saliva samples. For sample preparation, plasma or saliva was spiked with IS solution, and then ice-cold acetonitrile was added for protein precipitation. The sample was vortex-mixed and centrifuged. Next, the supernatant was evaporated under a gentle nitrogen stream, and the dry residue was reconstituted with water containing triethylamine (pH 6.0). For further cleaning of samples, MEPS was performed. First, C18 sorbent was activated with methanol and then ultrapure water. Next, the sample was drawn through the needle into the syringe and ejected, and three draw–eject cycles were applied on the same sample. The sorbent was washed with ultrapure water in order to remove matrix interferences; then, the analytes were eluted with methanol and diluted with ultrapure water. An aliquot of the final sample extract was injected into the HPLC system. The analysis was carried out on a C18 column with a mobile phase containing acetonitrile, water, *ortho*-phosphoric acid, and triethylamine (pH = 6.0). Detection was performed at 215 nm. The LOQ values obtained by the procedure were 0.1 μg/mL for both plasma and saliva samples. The authors obtained good correlation between the saliva and plasma lamotrigine concentrations, indicating possibilities for the application of saliva samples for TDM in routine clinical practice. The proposed analytical procedure was applied to the analysis of plasma and saliva samples from epilepsy patients treated with lomatrigine, and the results confirmed its usefulness for TDM. Concentration–time profiles of lamotrigine obtained from plasma and saliva samples collected at 2, 4, 8, and 12 h post-dose in two patients under oral lamotrigine therapy are presented in Figure 12. 

### 9.2. HPLC-FLD

Sometimes for the determination of antiepileptic drugs, a high-performance liquid chromatographic method with fluorescence detection was applied. Mercolini et al. described a HPLC-FLD method based on pre-column derivatization with dansyl chloride for the simultaneous determination of drugs used in monotherapy and in polytherapy for the treatment of different forms of epilepsy—gabapentin, vigabatrin and topiramate—in human plasma samples [132]. Plasma samples were prepared by SPE using mixed mode reversed-phase strong cation exchange cartridges. Cartridges were conditioned with methanol twice and equilibrated with ultrapure water twice. After addition to plasma samples of 0.1N HCl and IS solution, the resulting mixture was loaded onto a conditioned cartridge. Then, the cartridge was washed twice with 0.1N HCl, twice with a phosphate buffer at pH 5.0 and once with methanol. Then, the analytes were eluted with a mixture of ammonia, water, and acetonitrile. The eluate was evaporated to dryness, re-dissolved with ultrapure water, and subjected to a derivatization procedure. For derivatization to solution, the pretreatment plasma sample carbonate buffer at pH 10.5 and dansyl chloride solution were added. The mixture was vortexed and then kept at 50 °C for 10 min. The obtained solution was injected into the HPLC system. Investigated drugs were analyzed on a Hydro-RP column with a mobile phase containing acetonitrile and phosphate buffer at pH 5.3. The excitations and emission wavelengths were detected at 300 nm and 500 nm, respectively. LODs were established between 0.3 and 1.7 µg/mL, while LOQs were between 0.25 and 0.125 mg/mL. The proposed procedure was successfully applied for the determination of investigated antiepileptic drugs in plasma samples obtained from some epileptic and psychiatric patients.

### 9.3. LC-MS

LC-MS was relatively less often applied for the analysis of antiepileptic drugs compared to the analysis of drugs belonging to other groups of drugs. 

The antiepileptic drugs including levetiracetam, lamotrigine, zonisamide, topiramate, carbamazepine, phenytoin, divalproex sodium, oxcarbazepine, and the oxcarbazepine active metabolite 10,11-dihydro-10-hydroxycarbamazepine were determined in plasma by LC-MS/MS [129]. In the proposed procedure, plasma samples were spiced with IS and vortexed. Then, protein precipitation was performed by the addition of acetonitrile. Next, samples were centrifuged and the supernatant was diluted with acetonitrile/water (1:9, *v/v*). Such prepared samples were transfered into the HPLC system. The separation of analytes was achieved on a C18 column using a mobile phase containing acetonitrile, water, and ammonium acetate. Triple quadrupole MS with an ESI source was used for analytes detection. Carbamazepine, 10,11-dihydro-10-hydroxycarbamazepine, levetiracetam, lamotrigine, and oxcarbazepine were analyzed in positive ion mode, while phenytoin, topiramate, divalproex sodium, and zonisamide were analyzed in negative ion mode. The LLOQ = 5 ng/mL was obtained for levetiracetam, lamotrigine, carbamazepine, 10,11-dihydro-10-hydroxycarbamazepine, and oxcarbazepine; 10 ng/mL was obtained for zonisamide, topiramate, and phenytoin; 50 ng/mL was obtained for divalproex sodium. The authors applied the described procedure to determination of the drug concentration in 16 “generic brittleness” patients.

Additionally, examples of the application of liquid chromatography for the analysis of antiepileptic drugs are presented in Table 8.

## 10. Other Drugs

TDM is increasingly applied for other drugs such as anticoagulant, antidiabetic, iron-chelating drugs and many others. Chromatography with various detection methods was commonly used for this purpose.

### 10.1. HPLC-UV

In thalassaemic patients, iron overload, caused by the need for regular transfusions and increased gastrointestinal absorption can lead to iron accumulation in the body. This can cause damage to the liver, myocardium, spleen, and endocrine organs, inducing heart failure, diabetes, hypothyroidism, hypogonadism, and hepatic disease such as cirrhosis or liver cancer. Iron chelation therapy is used to reduce iron overload development due to its deposition in various organs such as liver and heart after regular transfusion. Therapeutic monitoring of chelation therapy in thalassemic patients is necessary to ensure effective treatment, compliance, and to avoid adverse side effects and toxicity. For the quantitation of oral iron chelating agent deferasirox in plasma samples of thalassaemic from patients, an HPLC-UV method was developed [139]. Samples were prepared by protein precipitation with solution of methanol and acetonitrile. After mixing, samples were centrifuged, and the obtained supernatant was injected into the HPLC system. Analysis was carried out on a C18 column with a mixture that consisted of methanol, acetonitrile, water, triethylamine, and orthophosphoric acid (pH = 9.3) as a mobile phase. The detection wavelength was set at 295 nm. The LOD obtained by the described procedure was 0.078 µg/mL, while the LOQ was 0.156 µg/mL. The correlation between deferasirox dose and its concentration in plasma was investigated. The obtained correlation was non-linear, which confirmed the necessity of using TDM for this drug.

Mattioli et al. also proposed an HPLC-DAD procedure for the quantification of deferasirox in human plasma [140]. HPLC analysis was performed on a C18 column using a mobile phase containing acetonitrile, methanol, water, disodium hydrogen phosphate, and an ion-pairing reagent: tetrabutylammonium hydrogen sulfate. The UV detection wavelength was set at 295 nm. The procedure was applied for the determination of deferasirox in plasma samples obtained from 80 patients with transfusion-dependent anemias, such as thalassemia.

Anticoagulants are drugs used for the treatment and prevention of thromboembolic diseases and for the prevention of stroke in patients with atrial fibrillation. These drugs have a narrow therapeutic range, and their effects depend on several factors, such as body weight, age, metabolism, diet, certain medical conditions, or the intake of additional drugs, among others. A higher dose may result in the risk of bleeding, while if it is too low, the risk of blood clot can increase. For these reasons, TDM for this group of drugs is indicated. Gouveia et al. applied the HPLC-DAD method for the simultaneous quantification of anticoagulant drugs in human plasma: apixaban, dabigatran, edoxaban, and rivaroxaban [141]. Before HPLC analysis, samples were prepared by protein precipitation followed by an SPE procedure carried out on Oasis PRIME HLB cartridges. After sample loading, cartridges were washed with water and a mixture of methanol and water. For elution of the investigated drugs and internal standard, methanol was used. The eluates were collected into glass tubes and dried to a solid residue under nitrogen. Then, the solid residue was reconstituted with a mixture of 0.1% formic acid in water and methanol and injected into an HPLC system. The separation of analytes was performed on a C18 column. A mixture of acetonitrile, water, and formic acid was applied as a mobile phase. A detector was set at 278 nm for the IS, 300 nm for dabigatran and apixaban, 289 nm for edoxaban, and 249 nm for rivaroxaban. Good separation selectivity for the investigated drugs and very symmetrical peaks of analytes were obtained by the proposed procedure. 

Histamine receptor antagonists encompass a group of chemically different compounds with the similar pharmacologic ability to competitively antagonize histamine at its receptors. These drugs block activation of the histamine receptors, which can stimulate sensory nerves and increase vascular permeability and mucus production. Blocking histamine receptors prevents the histamine contribution to the symptoms of allergic rhinitis: nasal itching and congestion, sneezing, rhinorrhea, and ocular irritation. Since the classic H1-receptor antagonists are not selective for the H1 site and cross the blood–brain barrier, they induce a variety of dopaminergic, serotonergic, and cholinergic responses. Therefore, monitoring their concentration in patients may be indicated in order to select the appropriate dose of these drugs. Fexofenadine is a non-sedating selective histamine H1 receptor antagonist. The drug is applied in the management of allergic rhinitis and chronic idiopathic urticaria. Fexofenadine was analyzed in human plasma for TDM and pharmacokinetic study by HPLC-UV [142]. The samples were extracted by the addition of diethyl ether. Then, they were shacked and centrifuged. The ether layer was evaporated in a water bath at 60 °C; then it was reconstituted with the mobile phase and injected into the chromatographic system. The UV detector was set at 215 nm for the fexofenadine and 330 nm for the IS (tinidazole). The LOD and LLOQ were 5 and 10 ng/mL, respectively. The developed procedure allowed for the determination of fexofenadine in the plasma samples obtained from patients treated with it.

Bezafibrate used to treat hyperlipidaemia was determined in human plasma by HPLC-UV [143]. Samples were prepared by the addition of IS, 0.1 N hydrochloric acid and tert-butyl methyl ether. Then, the samples were vortex-mixed and centrifuged. The supernatant was separated and evaporated to dryness under a nitrogen stream. The residue was reconstituted with a mixture of acetonitrile and water. For chromatographic analysis, C18 and a mobile phase containing acetonitrile, methanol, and phosphate buffer at pH 3.5 were applied. The UV detection was performed at λ = 230 nm. The LOD and LLOQ were 0.01 μg/mL and 0.05 μg/mL, respectively. The procedure was applied for the determination of bezafibrate in six healthy volunteers, who ingested a single oral dose of 200 mg.

### 10.2. HPLC-FLD

Salmeterol is a long-acting beta2-adrenergic receptor agonist drug that is used for the treatment of asthma and chronic obstructive pulmonary disease, as well as the prevention of exercise-induced asthma. The drug was determined by HPLC-FLD in less invasive dried blood spot samples from asthmatic patients [144]. In the procedure, a modified dispersive liquid–liquid microextraction (DLLME) technique by ionic liquids has been developed for sample preparation. An extraction solvent consisting of methanol, ionic liquid, and IS was added to the sample. Then, samples were sonicated and centrifuged. Next, each sample was withdrawn into a syringe and rapidly injected in 1 mL salinated (10% NaCl) and alkalized (pH = 12) water. A cloudy solution that consisted of very fine droplets of ionic liquid dispersed into aqueous sample was formed, and the analyte was extracted into the fine droplets. After centrifugation, the dispersed fine ionic liquid droplets settled to the bottom of the test tube. The sedimented phase was removed using a HPLC microsyringe and injected into the HPLC system. Analysis was performed on a C18 column with a mobile phase containing acetonitrile and ammonium acetate buffer at pH 6.0. Fluorescence detector operated at an excitation wavelength of 230 nm and emission wavelength of 340 nm. Low values of LOD (0.3 ng/mL) and LOQ (1.0 ng/mL) were obtained by the described procedure. The application of ionic liquid-based DLLME allowed the elimination of toxic organic solvents often used for extraction procedures.

Hydroxychloroquine, an old antimalarial drug, is also effective for the treatment of systemic lupus erythematosus and other autoimmunediseases. Its hematic concentration is closely related to the therapeutic response; therefore, monitoring the levels of the drug and its metabolites in patient’s blood helps the clinician in the evaluation of partial or complete unresponsiveness to treatment. Charlier et al. developed an ion-pairing LC-FLD method for the determination of hydroxychloroquine and its metabolites in whole blood: bisdesethylchloroquine, chloroquine, desethylchloroquine, and desethylhydroxychloroquine [145]. The same samples were also analyzed using a previously validated LC-MS/MS method. Before analysis, samples after the addition of IS in water were vortexed. Afterwards, 25% NH_3_ and diethyl ether were added. Then, the samples were vortexed and centrifuged. In the next step of sample preparation, the organic layer was air-dried for 14 h at room temperature. The residue was dissolved in mobile phase, centrifuged, and the supernatant was injected into a chromatographic system. Analytes were separated on a C18 column with a mobile phase containing acetonitrile, methanol, water, and ion-pairing reagent-sodium dodecyl sulfate. The pH of the mobile phase was adjusted to 9.4 with 25% NH_3_ and 25% orthophosphoric acid. FLD operating at excitation wavelength of 320 nm and emission wavelength of 370 nm was applied for the detection of investigated compounds. LC-MS/MS analysis was performed on a pentafluorophenyl column. A mixture that consisted of acetonitrile, water, and formic acid was applied in the procedure as a mobile phase. Analytes were detected using triple quadrupole MS with ESI operated in positive ionization mode. The LLOD obtained by LC-FLD was 1 ng/mL for all the analytes; the LLOQs were 15 ng/mL for desethylchloroquine and desethylchloroquine and 20 ng/mL in the case of hydroxychloroquine and bisdesethylchloroquine. The authors compared the results obtained by HPLC-FLD with LC/MS/MS data and concluded that the proposed procedure can be applied for routine clinical analyses.

Cisatracurium is a competitive neuromuscular blocker that acts by competing with acetylcholine for receptors on the motor end plate of the neuromuscular junctions to produce a blockade. Propofol acts as a short-acting anesthetic given intravenously to induce and also maintain general anesthesia. Both drugs are coadministered as a preoperative injection mixture. Ayad et al. proposed an HPLC-FLD method for the simultaneous determination of these drugs in human plasma [146]. Prior to chromatographic analysis, samples were prepared by protein precipitation with methanol. Separation was performed on a monolithic C18 column using a mobile phase containing methanol and phosphate buffer at pH 4.5. For the detection of analytes, excitation and emission wavelengths were set at 230 and 324 nm, respectively.

### 10.3. LC-MS

Dabigatran is a novel direct oral anticoagulant (DOAC) and a reversible inhibitor of thrombin that was determined in human plasma samples by LC-MS/MS [147]. Samples were prepared by liquid–liquid extraction after protein precipitation. HPLC separation was performed on a CN column with a mobile phase containing acetonitrile, water, and formic acid. The application of a CN column enables improving the sample resolution and avoiding the early elution of dabigatran previously seen when using a C18 column. For the detection of dabigatran electrospray ionization, time-of-flight MS was applied. The proposed method was successfully applied for the determination of dabigatran in plasma samples obtained from 30 patients to evaluate antithrombotic efficacy and anticoagulant activity of the drug following knee endoprosthesis surgery.

**Figure 13:**
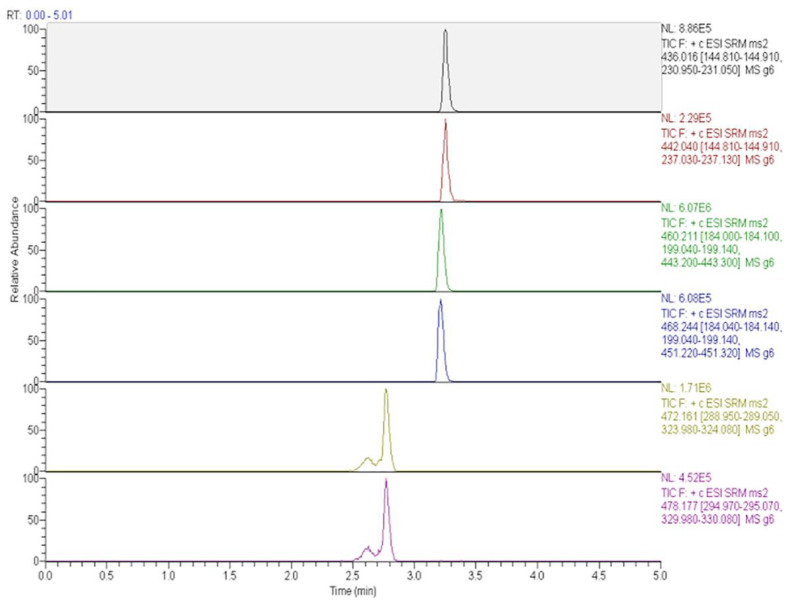
Representative chromatogram with retention times of dabigatran, ^13^C_6_-dabigatran, rivaroxaban, ^13^C_6_-rivaroxaban, apixaban, and ^13^C, ^2^H_7_ apixaban [148].

An HPLC-MS/MS method was developed for the simultaneous determination of direct oral anticoagulants apixaban, dabigatran, and rivaroxaban in human plasma samples [148]. Samples were prepared by the addition of methanol, vortex-mixed, centrifuged, and injected into HPLC system. Chromatographic analysis was performed on C18 with a mobile phase of methanol, water, and formic acid. Detection was performed by triple quadrupole MS with ESI operated in positive ionization mode. The representative chromatogram obtained for dabigatran, ^13^C_6_-dabigatran, rivaroxaban, ^13^C_6_-rivaroxaban, apixaban and ^13^C, ^2^H_7_ apixaban is presented in Figure 13.

Type 2 diabetes is a multifactorial disease and its treatment responsiveness is highly variable accordingly to the complexity of the pathogenic process. In the therapies of the disease, biguanides, first-generation sulfonylureas, second-generation sulfonylureas, meglitinides, a-glucosidase inhibitors, thiazolidinediones, DPP-4 inhibitors, glucagon-like peptide-1 receptor agonists, and sodium glucose co-transporter 2 inhibitors are used [149]. It is very important that the patient’s disease treatment includes a careful antidiabetic therapy selection, regarding the age, comorbidities, HbA1c levels, and the adverse effects associated with each active pharmaceutical ingredient. Therefore, to avoid the adverse effects, it is important to develop TDM, allowing therapeutic individualization.

**Figure 14:**
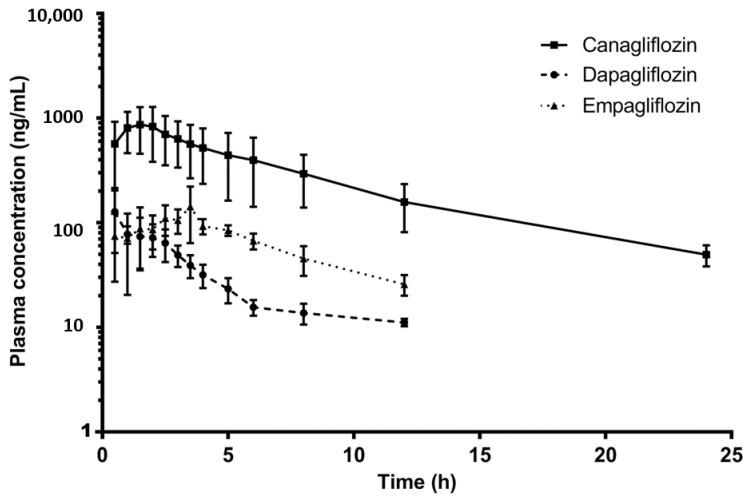
Gliflozin’s pharmacokinetic profile in association with metformin (1000 mg). The gliflozin doses were 100, 10, and 10 mg for canagliflozin, dapagliflozin, and empagliflozin, respectively. Data are presented as a mean ± standard deviation and n = 6 for each group [150].

Metformin and sodium–glucose cotransporter-2 inhibitors (canagliflozin, dapagliflozin, and empagliflozin) were simultaneously determined in human plasma by LC-MS/MS for pharmacokinetic and bioequivalence studies and TDM [150]. Before chromatographic analysis, samples were prepared by protein precipitation with acetonitrile containing 0.1% formic acid. After centrifugation, the supernatant was dried and then resuspended in a mixture containing acetonitrile, water, 1 mM ammonium formate, and 0.1% formic acid. Afterwards, samples were injected into the LC-MS/MS system for analysis. The separation of analytes was performed on a C18 column using a mobile phase containing acetonitrile, water, ammonium formate, and formic acid. Triple quadrupole MS with an ESI source operated in positive ionization mode was coupled to the chromatographic system for the detection of investigated drugs. The values of LOQ obtained by the proposed procedure were 25 ng/mL for canagliflozin and metformin, 15 ng/mL for empagliflozin, and 10 ng/mL for dapagliflozin. The pharmacokinetic profiles obtained by application of the described method of the sodium–glucose renal cotransporter 2 inhibitors inhibitors after coadministration with metfomin are presented in Figure 14.

A high inter-individual variability in response to treatment by antidementia drugs has been observed, which might partly be due to the high inter-individual variabilites in plasma concentrations. In elderly people, the presence of comorbidities and multiple comedication leading to drug–drug interactions, as well as genetic variations in metabolizing enzymes and transporters, might be causes of the observed inter-individual variabilities in plasma concentrations. For these reasons, the use of TDM in patients treated with anti-dementia drugs is indicated, although there are a few publications on this subject. 

Noetzli et al. proposed a UPLC-MS/MS method for the simultaneous determination of antidementia drugs, including the acetylcholinesterase inhibitors donepezil, galantamine, rivastigmine and its metabolite NAP 226-90, as well as *N*-methyl-d-aspartate (NMDA) receptor antagonist memantine, in human plasma [151]. In the sample preparation procedure, plasma samples were mixed with IS solution and acetonitrile, which was added for protein precipitation. Then, samples were vortex-mixed, sonificated, and centrifuged. The supernatants were evaporated to dryness, and the residues were reconstituted in the mobile phase at initial gradient conditions, vortex-mixed, again centrifuged, and transferred into a UPLC-MS/MS system. Chromatographic analysis was performed on a C18 column with a mobile phase containing acetonitrile and ammonium acetate buffer at pH 9.3. The chromatographic system was coupled to a tandem quadrupole MS equipped with an ESI operated in positive ionization mode. Results obtained by LC-MS and UPLC-MS/MS procedures were compared. The authors concluded that both methods are reliable and can be used for TDM in patients receiving antidementia drugs. However, the UPLC-MS/MS method is preferable with respect to specificity, sensitivity, and speed.

Lidocaine is applied as an anesthetic, sedative, antiarrhythmic, and anticonvulsant. As anticonvulsant is it also used in neonates. An LC-MS/MS method for the simultaneous quantification of lidocaine and its active metabolite monoethylglycinexylidide, which can accumulate and contribute to clinical toxicity, in plasma of neonates with seizures was developed and validated [152]. In the proposed procedure, before analysis, samples were prepared by a simple method of protein precipitation with methanol. Chromatographic separation was achieved on a C18 column using a mixture of acetonitrile, water, formic acid, and ammonium acetate. The detection of analytes was performed by triple quadrupole MS with ESI operated in positive ionization mode. The obtained LLOQ value was 0.2 mg/L for lidocaine and monoethylglycinexylidide. In the proposed procedure, a very small volume of plasma samples (only 10 µL) were required.

## 11. Protocols for Monitoring Drugs

Nowadays, most often, a large number of drugs are anlyzed in one biological sample during one chromatographic analysis. Many patients must be treated with multiple drugs at the same time. This can involve both the use of several drugs to treat the same disease or the treatment of several diseases occurring together in the same patient. The use of a multiple-drug regime can often lead to drug–drug interactions. Metabolites can provide additional information on the time of ingestion, the metabolic capacity, and compliance. Some metabolites, e.g., metabolites of nortryptyline, fluoxetine, and venlafaxine have also shared the primary biochemical actions of their parent drugs [11].

Analytical procedures for TDM should be established by determining the nature of the problem to be solved, selecting the appropriate matrix and methodology for sample preparation and analysis, validation of the analytical procedure, and interpretation of the obtained results. After administration, the drug penetrates into various tissues, and therefore, the appropriate type of sample to be collected for analysis should be selected. Enough sample should be selected given the concentration of the drug in the tissue to allow its detection; it may be the tissue in which the drug should exhibit its effect, and at the same time, it is advisable that the sampling should be as least invasive as possible. Sometimes, monitoring of the concentration of drug metabolite or metabolites, e.g., when the metabolite formed in the organism is an active form, is more advantageous and it must be taken into account in the selection of the sample’s kind. Most drugs easily penetrate into the blood where they obtain a characteristic concentration; therefore, most of the published procedures concern the determination of drugs in serum or plasma samples. These samples can also be relatively easy to prepare before analysis. 

Hydrophobic drugs partition into red cells, and the recommended sample is venous whole blood or finger prick capillary samples. The blood-sampling devices for therapeutic drug monitoring (TDM) program are applied also.

The type of sample matrix also partially determines the method of its preparation. During the sample preparation, at least protein and preferably other interfering substances from the sample should be removed before analysis. The choice of sample preparation technique relies on the chemical properties of analyzed drugs and/or metabolites, and achieving good recoveries of analytes is largely dependent on the polarity of the investigated compounds. The simplest and most often applied technique is protein precipitation. The technique can be easily automated and can be performed in 96-well plates, but samples prepared by the technique are relatively dirtier and diluted by the solvents used for precipitation, and this may lead to less sensitivity. The application of LLE is often the most effective way of removing matrix effects because ionized compounds, e.g., some phospholipids, do not partition readily into the organic phase. Hovewer, the method is time consuming and difficult to automate. 

The SPE columns available now cover a wide range of stationary phases—e.g., hydrophobic, ion exchange, and mixed mode stationary phases—which allows selecting the most optimal conditions for the extraction of analytes from a given matrix. SPE with an appropriate selection of extraction conditions allows obtaining very purified samples. The method can be easily automated and coupled on-line with a chromatographic system. 

Microextraction techniques such as solid-phase microextraction or dispersive liquid–liquid microextraction are also useful for biological sample preparation, especially when a small volume of samples are used. The choice of the sample preparation method and its conditions must always be considered taking into account the properties of the analytes and matrix, as well as the chromatographic method and detection method that will be used for the analysis.

The stationary phase should be selected both in terms of column chemistries and also consider the particle sizes of theadsorbent. More hydrophobic analytes posessing large molecules can be eaesly determined on hydrophobic alkylbonded) stationary phases (C18, C8). For more polar compounds having π–π bonds in their molecules, good results are obtained on the phenyl or cyanopropyl stationary phases. 

HILIC systems or by ion exchange chromatography are succesfully applied for the analysis of drugs with very small and polar molecules. 

Most mobile phases used for the analysis of drugs and their metabolites contained an organic modifier (acetonitrile or methanol), water, and the addition of acids (most often formic, acetic) or buffers at acidic pH. Rarely mobile phases containing addition of silanol blockers (mainly organic amines) or rarely ion-pair reagents were applied for the analysis of more polar compounds. On columns with smaller particle sizes, the better resolution is achieved with higher back pressure, and this could be a limiting factor in the choice of column. 

Currently, it is a trend to use sub-2 µm particles to increase peak resolution and sensitivity. The application of monolithic columns is also becoming more common. In comparison with conventional particulate columns, the monolithic columns may offer a shorter retention time, more robustness, and a better resolution of analytes. When MS detection is used, mobile phase additives must be compatible with MS. Only volatile additives such as ammonium formate, ammonium acetate, formic acid, trifluoroacetic acid, or ammonia can be applied.

Nowadays, LC-MS and especially LC-MS/MS have been established as the primary analytical technique to support TDM. LC-MS/MS combines the separation power of HPLC and specific measurement of charged ions by MS, which offer significantly higher sensitivity and specificity. Tandem mass spectrometers provide highly specific information for drug monitoring. The mass pairs of precursor and product ions which can be unique for any specific compounds. Comparison of the retention time of compounds in biological samples and the retention time of drug standards together with MS1 and MS2 information of tandem MS is more reliable than using the retention time of standard only. 

The literature collected in this article allows for the adaptation of the results and the creation of protocols for the determination of various combinations of drugs belonging to various groups.

## 12. Conclusions

TDM is a tool to improve the efficiency and safety of various drug therapies. The measurement of drug concentrations in human blood samples with the aim of obtaining optimal drug levels with the goal of achieving treatment efficacy is a valuable tool for personalizing therapeutic regimes and optimizing patient benefit. Although the efficacy is the most relevant aspect of treatment, medication safety must also be taken into consideration. 

TDM is increasingly used in therapy with various drugs, especially in the treatment by immunosuppressive, antibiotics, antivirals, antiepileptics, psychotropic, and anticancer drugs. Clinicians are increasingly employing TDM of various drugs to ensure adequate patient exposure.

For these reasons, efficient and reliable methods for the determination of various drugs and often their metabolites in biological samples are continuously required. Chromatography combined with selective detection techniques is a powerful analytical tool with superior specificity and sensitivity, which is used in a wide variety of TDM applications.

The first step in the chain of decisions regarding TDM is always the development and implementation of reliable analytical methods, which should be suitable for the repeated determination of drug in biological samples over long periods of time.

Drug concentrations are routinely measured in serum or plasma samples. These matrices are highly suited to investigating the relationship between efficacy and drug levels in most cases. Increasingly, some drugs are determined in saliva samples, whose collection is non-invasive and painless. The sensitivity of chromatographic techniques has enabled the investigation of drug concentrations in alternative matrices, including dried blood spots, peripheral blood mononuclear cells, bile, and breast milk.

Biological samples before chromatographic analysis were prepared by various procedures, most often protein precipitation, LLE, and SPE. Nowadays, microextraction by packed sorbent is most often applied for biological samples preparation. Samples analyzed by HPLC-UV often require extensive sample preparation. As a result of the sensitivity and selectivity of the LC-MS/MS, extensive sample preparation techniques such as SPE and LLE are often unnecessary. Sometimes, a step of sample preparation is automated and coupled with chromatographic systems. Still, a main weakness of LC-based analytical procedures is their frequent lack of an automated sample preparation step before analysis.

Methods based on liquid chromatography coupled to mass spectrometry and especially tandem mass spectrometry are becoming increasingly popular due to their high specificity, sensitivity, and the possibility for the simultaneous quantification of different drugs and metabolites. MS technologies are continuously evolving with new ion source designs, enhancement of resolution and sensitivity, and miniaturization. Most of the currently applied MS methods use triple quadrupoles as mass analyzers. The classic ionization methods, electron ionization and chemical ionization, are not found to be currently used much for TDM because of their limitations in analyzing the wide range of polar compounds to which most drugs belong. Nowadays, most of the MS applications use matrix-assisted laser desorption/ionization, atmospheric pressure chemical ionization, and especially electrospray ionization. Electrospray ionization is one of the most popular atmospheric pressure ionization processes employed in clinical and research laboratories due to its ease of use. The positive ionization mode was used for drugs detection in most cases. 

Liquid chromatography with ultraviolet detection is another chromatographic technique that has been used for therapeutic drug monitoring. The advantages of UV detection are its ruggedness, ease of use and cost-efficiency mean. For those reasone, UV detection is often applied in many development methods for the analysis of various drugs in biological samples, especially whose body concentrations are relatively high. However, HPLC-UV often requires extensive sample preparation due to the limited specificity of the detection mode and the poor ultraviolet absorbance of some compounds.

HPLC methods with fluorescence and electrochemical detection for the monitoring of analytes offering very low detection limits are also promising tools for the quantification of some drugs and their metabolites for TDM purposes.

The optimization of chromatographic analysis parameters is the key to obtaining the best separation of analytes and successful analysis. The different controlling factors for the best separation include the choice of stationary phase, composition of the mobile phase, its pH, flow rate, and temperature. The selection of suitable stationary and mobile phases is most important for the successful analytical process of determining the concentration of drugs in biological samples. In most procedures, analytes were separated on alkylbonded stationary phases—most often C18 and rarely C8. Increasingly, phenyl stationary phases are used for the separation of some drugs and their metabolites, especially with basic properties. 

The composition of the mobile phase was usually dependent on the properties of the analytes and the stationary phase used. Most often for drug analysis, mobile phases contained an organic modifier (acetonitrile or methanol), water, and the addition of acids (most often formic or acetic, rarely phosphoric) or buffers at acidic pH. Sometimes, the addition of ion-pairing reagents or organic amines applied as silanol blockers (most often triethylamine) were used for the analysis of basic drugs.

Thus, future efforts should concentrate on strategies aiming to maximize the potential therapeutic benefit of patients therapies by optimizing dosage regimens with TDM in a personalized medicine setting.

The application of hyphenation of solid phase microextraction–liquid chromatography–tandem mass spectrometry to achieve detections at nanogram and picogram levels is the future direction for analytical methods for TDM.

## Figures and Tables

**Table 1:** Immunosuppressive drugs. ESI: electrospray ionization, LOD: limit of detection, LOQ: limit of quantification.

Investigated Drugs	Matrix	Method of Sample Preparation	Stationary Phase (Column)	Mobile Phase	Detection	LOD	LOQ	Ref.
Mycophenolic acid	Plasma	Ultrafiltration	C18	Acetonitrile, water, and HCl (pH 4.3–4.7)	UV at 254 nm	-	-	[25]
Mycophenolic acid	Plasma	Protein precipitation with 10% acetic acid and ultrafiltration	C18	Methanol, water, formic acid, and ammonium acetate	Triple quadrupole MS with ^+^ESI	-	-	[26]
Mycophenolic acid and its major metabolites, mycophenolic acid, glucuronide, and mycophenolic acid acyl-glucuronide	Plasma Urine	Hemolysis with zinc sulfate and protein precipitation with methanol was performed in 96-well format	C18	Methanol, water, and formic acid	Triple quadrupole MS with +ESI	-	0.097 µg/mL	[27]
Everolimus	Whole blood	Protein precipitation with acetonitrile	C18	Methanol, water, formic acid, and ammonium acetate	Triple quadrupole MS with ESI	-	1.7 ng/mL	[28]
Everolimus, sirolimus, tacrolimus, cyclosporin A, and mycophenolic acid	Whole blood or plasma (for mycophenolic acid)	Hemolysis with zinc sulfate and protein precipitation with acetonitrile (for plasma only protein precipitation with acetonitrile)	C18	Methanol, water, formic acid, and ammonium acetate	Triple quadrupole MS with ^+^ESI	0.5 ng/mL for everolimus, sirolimus, and tacrolimus, 25 ng/mL for cyclosporin A, and 100 ng/mL for mycophenolic acid	-	[19]
Ciclosporin, everolimus, sirolimus, tacrolimus	Whole blood	Sample preparation was performed in 96-well format. Plate was vigorously shaken on a vortex mixer and then samples were centrifuged	C18	Methanol, water, formic acid, and ammonium formate	Triple quadrupole MS with ^+^ESI	-	0.2 ng/mL for everolimus, sirolimus, and tacrolimus and 3 ng/mL for ciclosporin	[20]
Cyclosporin A, sirolimus, tacrolimus	Whole blood	Hemolysis with zinc sulfate and protein precipitation with methanol	First column: Cyclone-P TurboFlow colum and second column: C8	First eluent: Acetonitrile, isopropanol, and acetone, second eluent: methanol, water, formic acid and ammonium formate	Triple quadrupole MS with ^+^ESI	-	12 mcg/L for cyclosporine A, 1.0 mcg/L for tacrolimus, and 1.1 mcg/L for sirolimus	[29]
Cyclosporine A, tacrolimus, sirolimus, and everolimus	Whole blood	Hemolysis with zinc sulfate and protein precipitation with methanol	Phenyl Hexyl	methanol, water, formic acid, acetic acid and ammonium acetate	Triple quadrupole MS with ^+^ESI	-	26.6 ng/mL for cyclosporine A, 1.13ng/mL for everolimus, 1.57 ng/mL for sirolimus, and 1.28 ng/mL for tacrolimus	[30]
Tacrolimus	Whole blood	Hemolysis with zinc sulfate was performed in 96-well format	C18	Methanol, water, formic acid, ammonium acetate	Triple quadrupole MS with ^+^ESI	-	1.0 µg/L	[31]

**Table 2:** Anticancer drugs. LLE: liquid–liquid extraction, SPE: solid-phase extraction.

Investigated Drugs	Matrix	Method of Sample Preparation	Stationary Phase (Column)	Mobile Phase	Detection	LOD	LOQ	Ref.
Methotrexate	Plasma	Protein precipitation with acetonitrile and then LLE with chloroform and methylene chloride	C18	Acetonitrile, water, acetic acid, and sodium acetate (pH 4.0)	UV at 305 nm	-	0.10 μM	[43]
Methotrexate	Serum	SPE on SPE LC-Ph cartridges with phenyl stationary phase. Cartridges were preconditioned with methanol followed with water. After sample loading, cartridges were washed with 5% methanol in 0.01 N HCl and eluted with methanol	C18	Acetonitrile and sodium acetate buffer (pH 3.6)	UV at 307 nm	0.003 mM	0.01 mM	[44]
Ibrutinib	Plasma	SPE on Oasis HLB cartridges. For washing, water and 60% methanol in water (*v/v*) were applied. For analytes elution, methanol was used to dryness at 80 °C using a rotary evaporator. The dried residues were reconstituted in 50 μL methanol, and a 30 μL aliquot was injected into the HPLC system.	C18	Acetonitrile, water, and monopotassium phosphate (pH 3.0)	UV at 260 nm	-	10 ng/mL	[45]
Mitotane	Plasma	Protein precipitation with methanol	C8	Acetonitrile, potassium hydrogen phosphate, orthophosphoric acid, and tetramethylammonium (pH 3)	UV at 230 nm	0.1 mg/L	0.78 mg/L	[46]
Mitotane and its metabolites: o,p’-DDE (dichlorodiphenylethene) and o,p’-DDA (dichlorodiphenylacetate)	Plasma	LLE with acetone for the extraction of mitotane and o,p’-dichlorodiphenyldichloroethylene (o,p’-DDE) and LLE with a mixture of methanol and acetonitrile for the extraction of o,p’-DDA	C18	Acetonitrile, methanol, and water for analysis of mitotane and o,p’-DDE and acetonitrile, methanol, waterand triethylamine for o,p’-DDA	UV at 218 nm for mitotane and o,p’-DDE and UV at 235 nm for o,p’-DDA	-	-	[47]
Imatinib, dasatinib, and nilotinib	Plasma	SPE on C18 cartridges	C18	Acetonitrile, methanol, water, and triethylamine	UV at 267 nm	10 ng/mL for imatinib and nilotinib and 50 ng/mL for dasatinib	50 ng/mL for imatinib and nilotinib and 100 ng/mL for dasatinib	[48]
Tamoxifen	Plasma	LLE with hexane and n-propanol and re-extraction with aqueous solution of 0.1% (*v/v*) phosphoric acid	C18	Acetonitrile and triethylammonium phosphate buffer at pH 3.3	UV at 280 nm	-	from 0.75 to 8.5 ng ml^−1^	[49]
Hydroxyurea	Plasma	Protein precipitation with methanol	C18	Acetonitrile, water, and ammonium acetate	UV at 240 nm	-	5 μM	[50]
Imatinib, dasatinib and nilotinib	Peripheral blood mononuclear cell	LLE with a mixture of acetonitrile and methanol	C18	Acetonitrile, water, and formic acid	Quadrupole MS with ^+^ESI	0.06 ng	0.25 ng	[51]
Dasatinib, erlotinib, gefitinib, imatinib, lapatinib, nilotinib, sorafenib, sunitinib	Plasma	Protein precipitation with acetonitrile	C18	Methanol, water, and ammonium hydroxide (pH 10.5)	Triple quadrupole MS with ^+^ESI	-	-	[52]
5-fluorouracil and its metabolites	Plasma	LLE with ethyl acetate and 2-propanol	C18	Methanol, water, ammonium acetate, and formic acid	Triple quadrupole MS with ^+^ESI	-	0.1 µM	[53]
1-β-d-Arabinofuranosylcytosine 1-β-d-arabinofuranosyluracil	Plasma urine	Protein precipitation with acetonitrile	C18	Acetonitrile and water or methanol, water, and formic acid	Triple quadrupole MS with ^+^ESI	-	1.0 ng/mL for 1-β-d-arabinofuranosyluracil and 5.0 ng/mL for 1-β-d-arabinofuranosylcytosine	[54]
Rituximab	Serum	Vortexing	C3	Acetonitrile, 2-propanol, water, and formic acid	Triple quadrupole time-of-flight MS with ^+^ESI	-	-	[55]

**Table 3:** Antibiotics.

Investigated Drugs	Matrix	Method of Sample Preparation	Stationary Phase (Column)	Mobile Phase	Detection	LOD/LLOD	LOQ/LLOQ	Ref.
Hydroxychloroquine, minocycline, and doxycycline	Serum	LLE with diethyl ether for the extraction of hydroxychloroquine and LLE with mixture of ascorbic acid, phosphate sulfite, and ethyl acetate for the extraction of minocycline and doxycycline	Phenyl	1% triethylamine and 1 mM oxalic acid in water adjusted to pH 2.4 with orthophosphoric acid at 85%	UV at 343 nm	-	LLOQ was 0.25 μg/mL for hydroxychloroquine, 1.25 μg/mL for minocycline, 1.25 μg/mL for doxycycline	[66]
Ertapenem	Plasma	LLE with acetonitrile and then with dichloromethane	C18	Acetonitrile, water, and ammonium acetate at pH 4.5	UV at 254 nm and ion trap-time of flight mass spectrometer (IT-TOF) MS with ^-^ESI for the detection of ertapenem and ^+^ESI for IS (meropenem)	0.1 mg/mL and 0.05 mg/mL for the HPLC-UV and LC-MS methods, respectively	0.5 mg/mL for HPLC-UV and LC-MS methods	[67]
Latamoxef	Plasma	Protein precipitation with methanol	C18	Acetonitrile, water, ammonium acetate, and acetic acid (pH 6.5)	UV at 275 nm	-	3.0 μg/mL	[68]
Meropenem	Serum	SPE on Strata-X polymeric RP cartridges	C18	Methanol and tris(hydroxymethyl)aminomethane buffer (pH 8.5)	UV at 300 nm	-	3.5 mg/L	[69]
Levofloxacin, pefloxacin, ciprofloxacin, and moxifloxacin	Plasma	Protein precipitation with methanol	C18	Acetonitrile, methanol, water, and phosphoric acid	UV at 293 nm	-	0.05 mg/L for pefloxacin and ciprofloxacin, 0.125 mg/L for levofloxacin, and 0.1 mg/L for moxifloxacin	[70]
Vancomycin	Plasma	Protein precipitation with acetonitrile	C18	Methanol, ammonium acetate, and formic acid buffer (pH 4.0)	UV at 240 nm	-	1 μg/mL	[71]
Piperacillin, meropenem, ceftazidime, and flucloxacillin	Whole blood	Protein precipitation with acetonitrile	C18	Acetonitrile, water, and phosphoric acid	UV at 210 nm, 230 nm, 260 nm, and 306 nm	-	For piperacillin, ceftazidime and flucloxacillin was 5 µg/ml, for meropenem, it was 2 µg/ml	[72]
Rifampicin	Plasma	SPE on Oasis HLB columns	C8	Acetonitrile and acetate buffer (pH 5.7)	UV at 335 nm	-	0.05 mg/L	[73]
Biapenem	Peritoneal fluid and bile	Centrifugation	C18	Acetonitrile and sodium phosphate buffer (pH 7.4)	UV at 300 nm	0.01 μg/mL in peritoneal fluid for meropenem and biapenem and 0.02 μg/mL in bile samples	0.05 μg/mL for both investigated drugs were in peritoneal fluid and 0.1 μg/mL in bile samples	[74]
Linezolid	Plasma saliva	Vortexed and centrifuged with sodium chloride solution and ethyl acetate	C18	Acetonitrile, tetrahydrofuran, and ammonium acetate buffer (pH 4.4)	UV at 254 nm	-	0.13 μg/mL	[75]
Temocillin	Serum	Protein precipitation with methanol and ultrafiltration	Phenyl	Acetonitrile, water, and formic acid	Tandem quadrupole MS ^+^ESI	0.1 mg/L and (total concentration) 0.05 mg/L (unbound concentration)	1 mg/L and (total concentration) 0.5 mg/L (unbound concentration)	[76]
Cefoperazone and sulbactam	Serum	Protein precipitation with acetonitrile	C18	Acetonitrile, water, and formic acid	Triple quadrupole MS with ESI	0.03 μg/mL for sulbactam, and 0.01 μg/mL for cefoperazone	-	[77]

**Table 4:** Antifungal drugs.

Investigated Drugs	Matrix	Method of Sample Preparation	Stationary Phase (Column)	Mobile Phase	Detection	LOD	LOQ	Ref.
Voriconazole	Serum	LLE with a mixture of hexane and dichloromethane	C8	Acetonitrile and sodium potassium phosphate buffer at pH 6.0	UV at λ = 250 nm	0.25 mg/L	0.5 mg/L	[84]
Posaconazole	Serum		C18	Acetonitrile and water	UV at 262 nm	-	0.125 lg/mL	[85]
Isavuconazole, fluconazole, itraconazole, voriconazole, posaconazole	Plasma	Protein precipitation with acetonitrile and centrifugation	C18	Acetonitrile, water, and 0.05 % formic acid	Single-quadrupole ESI^+^MS	7.81 ng/mL for fluconazole, 14.65 ng/mL for voriconazole, 7.81 ng/mL for posaconazole, itraconazole, and isavuconazole	-	[86]
Isavuconazole, voriconazole, posaconazole, fluconazole, caspofungin, flucytosine, itraconazole, and its metabolite OH-itraconazole	Plasma	Protein precipitation with acetonitrile	C18	Acetonitrile, water, and 0.1 % formic acid	Single-quadrupole ESI^+^MS	-	From 0.2 mg/Lto 2 mg/L	[87]
Voriconazole	Plasma	Protein precipitation with methanol	C18	Acetonitrile, water, and 0.1 % formic acid	Triple quadrupole MS with ^+^ESI	-	2.49 ng/mL	[88]
Itraconazole	Plasma	Protein precipitation with acetonitrile	C18	Acetonitrile, water, and formic acid	Qaadrupole MS with ^+^ESI	0.015 μg/mL	0.031 μg/mL	[89]
Voriconazole, posaconazole, fluconazole, and itraconazole	Serum	LLE with acetonitrile and formic acid	C8	Acetonitrile, water, ammonium formate, and formic acid	Triple quadrupole MS with ^+^ESI	0.005 μg/mL for voriconazole, 0.01 μg/mL for posaconazole and itraconazole, and 0.1 μg/mL for fluconazole	0.01 μg/mL for voriconazole, 0.02 μg/mL for posaconazole and itraconazole, and 0.2 μg/mL for fluconazole	[90]
Fluconazole, posaconazole, itraconazole, voriconazole, and hydroxyitraconazole	Serum	Protein precipitation a mixture of cyanoimipramine, methanol, and acetonitrile	C18	Acetonitrile, water, acetic acid, ammonium acetate, and trifluoroacetic anhydride	Triple quadrupole MS with ^+^ESI	0.53 mg/L for fluconazole, 0.11 mg/L for posaconazole and itraconazole, and 0.10 mg/L for voriconazole and hydroxyitraconazole	-	[91]

**Table 5:** Antiviral drugs.

Investigated Drugs	Matrix	Method of Sample Preparation	Stationary Phase (Column)	Mobile Phase	Detection	LOD	LOQ	Ref.
Elvitegravir, dolutegravir, and rilpivirine	Plasma	SPE on Oasis HLB cartridges conditioned with methanol followed by phosphate buffer at pH 3.23, washed with phosphate buffer and eluted with a mixture of methanol and acetonitrile	C18	Acetonitrile, water, orthophosphoric acid, and KH_2_PO_4_ (pH = 3.23)	UV at 265 nm for dolutegravir and elvitegravir, 290 nm for rilpivirine	-	62.5 ng/mL for elvitegravir, 125 ng/mL for dolutegravir and 39.06 ng/mL for rilpivirine	[95]
Telaprevir	Plasma	SPE on Oasis HLB cartridges	C18	Acetonitrile, methanol, ammonium acetate buffer at pH 8.0	UV at 276 and 286 nm	156.25 ng/mL	312.5 ng/mL	[96]
Saquinavir, atazanavir, amprenavir, darunavir, lopinavir, ritonavir, etravirine, efavirenz, and nevirapine	Dried plasma spot	Thermal inactivate HIV, then extraction with a mixture of tert-butylmethylether and NH_3_	C18	Acetonitrile, water, and formic acid (gradient elution)	Single quadrupole with ESI^+^MS except efavirenz, which was detected in ESI^-^ mode	From 4.9 ng/mL to 29.3 ng/mL	From 11.7 ng/mL to 58.6 ng/mL	[97]
Abacavir, atazanavir, bictegravir, cobicistat, darunavir, dolutegravir, efavirenz, elvitegravir, emtricitabine, etravirine, lamivudine, nevirapine, raltegravir, rilpivirine, ritonavir, and tenofovir	Plasma	Vortexing and centrifugation	C18	Acetonitrile, water, and formic acid (gradient elution)	Triple quadrupole MS with ESI^+^ except for efavirenz, which was detected in ESI^-^ mode	-	From 2.5 ng/mL to 5 ng/mL	[98]
Lamivudine, lopinavir, ritonavir, and zidovudine	Plasma	SPE on Oasis HLB cartridge	C18	Acetonitrile, water, and formic acid	TOF-MS/MS	-	0.47 ng/mL for lamivudine, 0.28 ng/mL for lopinavir, 0.30 ng/mL for ritonavir, and 0.66 ng/mL for zidovudine	[99]

**Table 6:** Cardiovascular drugs.

Investigated Drugs	Matrix	Method of Sample Preparation	Stationary Phase (Column)	Mobile Phase	Detection	LOD	LOQ	Ref.
Aorvastatinin	Serum	LLE with ethyl acetate	C18	Methanol and phosphate buffer at pH 3.5	UV at 247 nm	1.2 ng/mL	3.0 ng/mL	[107]
Milrinone	Plasma	SPE on C18 cartriges	C18	Methanol, water, and ammonium acetate	Triple quadrupole MS with ^+^ESI	-	2.4 ng/mL	[108]
Aliskiren, prasugrel, and rivaroxaban	Urine	MEPS was performed using a 250 µL volume syringe coupled with a barrel insert and needle assemblies packed with the different sorbent (C2, C8, C18, and M1 containing 80% C8 and 20% SCX)	C18	Acetonitrile, water, and formic acid	Triple quadrupole MS with ^+^ESI	-	5.0 pg/mL for rivaroxaban and aliskiren and 0.5 pg/mL for prasugrel	[109]

**Table 7:** Psychotropic drugs. FLD: fluorescence detection.

Investigated Drugs	Matrix	Method of Sample Preparation	Stationary Phase (Column)	Mobile Phase	Detection	LOD	LOQ	Ref.
Hydroxybupropion, the major active metabolite of bupropion	Serum	Protein precipitation with acetonitrile and SPE on CN cartridges	CN	Acetonitrile and dipotassium hydrogen phosphate (pH 6.4)	UV at 214 nm	-	100 ng/mL	[120]
Licarbazepine	Serum	Protein precipitation with methanol	C18	Acetonitrile, water, and sodium dihydrogen phosphate monohydrate	UV at 306 nm	0.0182 μg/mL	0.20 μg/mL	[121]
Sulpiride	Plasma	Centrifugation	C18	Acetonitrile, water, phosphoric acid, ammonium dihydrogen orthophosphate	FLD λ = 300 nm for excitation and λ = 365 nm for emission	1 ng/mL	2 ng/mL	[122]
Fluoxetine, its metabolite norfluoxetine, and paroxetine	Plasma	Microextraction by packed sorbent (MEPS) on C8 MEPS cartridge activated with methanol and conditioned ultrapure water. After sample loading, cartridges were washed with 5% ammonium hydroxide aqueous solution, and the analytes were eluted with a mixture containing methanol and 1% of formic acid.	C18	Acetonitrile, methanol, water, sodium phosphate monobasic anhydrous and dipotassium hydrogen phosphate anhydrous (pH 3.0)	FLD at λ = 240 nm (excitation) and 312 nm (excitation) for fluoxetine and norfluoxetine and λ = 295 nm (excitation) and 350 nm (excitation) for paroxetine	5 ng/mL for fluoxetine and norfluoxetine and 1 ng/mL for paroxetine	20 ng/mL for fluoxetine and norfluoxetine and 5 ng/mL for paroxetine	[123]
Amisulpride, asenapine, desmethyl-mirtazapine, iloperidone, mirtazapine, norquetiapine, olanzapine, paliperidone, quetiapine, and risperidone	Plasma	Protein precipitation with acetonitrile	C18	Acetonitrile and ammonium formate buffer pH 3.0	Tandem quadrupole MS with ESI^+^	-	0.5 ng/mL for asenapine, desmethyl-mirtazapine, iloperidone, mirtazapine, olanzapine, paliperidone, and risperidone and 1 ng/mL for amisulpride, norquetiapine, and quetiapine	[124]
Olanzapine, quetiapine, clozapine, haloperidol, chlorpromazine, mirtazapine, paroxetine, citalopram, sertraline, imipramine, clomipramine, fluoxetine, carbamazepine, lamotrigine, diazepam, and clonazepam	Plasma	Protein precipitation with acetonitrile	C18	Acetonitrile, water, ammonium acetate, and formic acid	Tandem quadrupole MS with ESI^+^	-	From 0.2 ng/mL to 5.0 ng/mL	[125]
7-Hydroxy-N-desalkyl-quetiapine, 7-hydroxy-quetiapine amisulpride, aripiprazole, risperidone, paliperidone, bromperidol, clozapine, haloperidol, N-desmethylclozapine, N-desmethylolanzapine, olanzapine, pipamperone, zuclopenthixol	Oral fluid	LLE with methyl tert-butyl ether	C18	Acetonitrile, water and ammonium acetate at pH 3.7	Triple quadrupole with ESI^+^	-	From 0.8 to 60 ng/mL	[126]
Chlorpromazine, haloperidol, zuclopenthixol, clozapine, risperidone, quetiapine, aripiprazole, olanzapine and some active metabolites (dehydroaripiprazole, N-desmethylclozapine, and 9-hydroxyrisperidone)	Serum	Protein precipitation with methanol	C18	Acetonitrile, water and formic acid	Triple quadrupole with ESI^+^	-	From 0.5 to 1.0 ng/mL	[127]

**Table 8:** Antiepileptic drugs.

Investigated Drugs	Matrix	Method of Sample Preparation	Stationary Phase (Column)	Mobile Phase	Detection	LOD	LOQ	Ref.
Rufinamide and zonisamide	Plasma	Protein precipitation with acetonitrile	C18	Acetonitrile, methanol, and potassium dihydrogen phosphate buffer at pH 4.5	UV at 210 nm	0.5 µg/mL for both analytes	2.0 µg/mL for both analytes	[133]
Zonisamide	Serum	Protein precipitation with methanol	C18	Isopropyl alcohol, acetonitrile, water, and acetic acid	UV at 238 nm	-	0.94 μg/mL	[134]
Carbamazepine, lamotrigine, oxcarbazepine, phenobarbital, phenytoin, and the active metabolites carbamazepine-10,11-epoxide and licarbazepine	Plasma	Protein precipitation with acetonitrile and microextraction by packed sorbent	C18	Acetonitrile, methanol, water, triethylamine, and ortho-phosphoric acid (pH 6.5)	UV at 215, 237, and 280 nm	From 0.015 to 0.09 µg/mL	From 0.1 to 0.4 µg/mL	[128]
Lacosamide	Serum saliva	Centrifugation after addition of perchloric acid (60%)	C18	Methanol, water and formic acid	UV at 215 nm	-	for serum 1 µmol/L (0.25 mg/L), for saliva 0.05 lmol/L (0.01 mg/L)	[135]
Perampanel	Plasma	LLE with diethyl ether	C18	Acetonitrile, water, sodium acetate, and acetic acid	FLD excitation λ = 290 nm, emission λ = 430 nm	-	1 ng/mL	[136]
Carbamazepine, oxcarbazepine, and active metabolite eslicarbazepine	Plasma and serum	Protein and phospholipids precipitation with acetone	C18	Acetonitrile, methanol, water, and formic acid	Triple quadrupole MS with ^+^ESI	-	-	[137]
Valproic acid and its metabolites: 2-propyl-4-pentenoic acid, 2-propyl-2,4-pentadienoic acid and 2-propyl-2-pentenoic acid	Serum	SPE on Oasis HLB cartridges pre-conditioned by methanol, followed by water. After loading of samples, cartridges were washed with methanol and eluted with a mixture containing methanol and ammonium acetate	C18	Acetonitrile, water, and ammonium acetate	Triple quadrupole MS with ^-^ESI	-	1 μg/mL for valproic acid, 0.5 μg/mL for 2-propyl-2-pentenoic acid, 10 ng/mL for 2-propyl-4-pentenoic acid and 25 ng/mL for 2-propyl-2,4-pentadienoic acid	[138]

## Abbreviations

TDM, therapeutic drug monitoring. ***Chromatographic methods:*** LC, liquid chromatography; HPLC, high performance liquid chromatography; UPLC, utra performance liquid chromatography. ***Detection techniques:*** UV, ultraviolet detection; UV-Vis; ultraviolet – visible detection; DAD, diode-array detection; PDA, photodiode-array detection; FLD, fluorescence detection; ED, electrochemical detection; MS, mass spectrometry; MS/MS, tandem mass spectrometry; HRMS, high-resolution mass spectrometry; TOF, time-of-flight; QqQ-TOF, triple quadrupole time-of-flight; ESI, electrospray ionization; MRM, multiple reaction monitoring. ***Extraction techniques:*** DLLME, dispersive liquid–liquid microextraction; LLE, liquid-liquid extraction; SPE, solid-phase extraction; SPME, solid-phase microextraction; MEPS, microextraction with packed sorbent; SBSE; stir bar sorptive extraction. ***Qualitative and quantitative analysis:*** LOD, limit of detection; LOQ, limit of quantification; LLOQ, lower limit of quantification; IS, internal standard. ***Others***: 1-D, one-dimensional; 2-D, two-dimensional.
